# Comparative molecular evolution of chitinases in ascomycota with emphasis on mycoparasitism lifestyle

**DOI:** 10.1099/mgen.0.000646

**Published:** 2021-09-13

**Authors:** Chao Wang, Zhao-Qing Zeng, Wen-Ying Zhuang

**Affiliations:** ^1^​ State Key Laboratory of Mycology, Institute of Microbiology, Chinese Academy of Sciences, Beijing 100101, PR China; ^2^​ University of Chinese Academy of Sciences, Beijing 100049, PR China; ^3^​ Institute of Fundamental and Frontier Sciences, University of Electronic Science and Technology of China, Chengdu 610054, PR China

**Keywords:** ascomycota chitinases, episodic selection, mycoparasitism, phylogeny

## Abstract

Chitinases are involved in multiple aspects of fungal life cycle, such as cell wall remodelling, chitin degradation and mycoparasitism lifestyle. To improve our knowledge of the chitinase molecular evolution of Ascomycota, the gene family of 72 representatives of this phylum was identified and subjected to phylogenetic, evolution trajectory and selective pressure analyses. Phylogenetic analysis showed that the chitinase gene family size and enzyme types varied significantly, along with species evolution, especially for groups B and C. In addition, two new subgroups, C3 and C4, are recognized in group C chitinases. Random birth and death testing indicated that gene expansion and contraction occurred in most of the taxa, particularly for species in the order Hypocreales (class Sordariomycetes). From an enzyme function point of view, we speculate that group A chitinases are mainly involved in species growth and development, while the expansion of genes in group B chitinases is related to fungal mycoparasitic and entomopathogenic abilities, and, to a certain extent, the expansion of genes in group C chitinases seems to be correlated with the host range broadening of some plant-pathogenic fungi in Sordariomycetes. Further selection pressure testing revealed that chitinases and the related amino acid sites were under positive selection in the evolutionary history, especially at the nodes sharing common ancestors and the terminal branches of Hypocreales. These results give a reasonable explanation for the size and function differences of chitinase genes among ascomycetes, and provide a scientific basis for understanding the evolutionary trajectories of chitinases, particularly that towards a mycoparasitic lifestyle.

## Data Summary

We confirm that all supporting data, code and protocols have been provided within the article or through Supplementary Material. Supplementary figures are provided with this article and are available online, while the Supplementary tables and text documents can be download at: http://lab.malab.cn/~wangchao/supplementary/.

**Impact Statement**

Chitinases are involved in multiple aspects of fungal life cycle. The chitinase genes from 72 representative species of Ascomycota were identified and subjected to phylogenetic, evolution trajectory and selective pressure analyses. Our results showed that expansions and contractions of chitinase genes occurred several times during evolution, especially for mycoparasitic species. The biological functions of the chitinase groups are different, with group B chitinases being correlated with ascomycete lifestyle evolution, while group C chitinases might relate to host range broadening. It was also demonstrated that chitinases and their related amino acids sites have been under episodic positive selection during Ascomycota evolution, especially at the nodes and branches leading to Hypocreales. This suggests that the chitinase family underwent a diversified evolutionary trajectory and plays an essential role in species trophic style transformation, particularly evolution towards a mycoparasitic style.

## Introduction

Chitin is the second most abundant polysaccharide in nature and a major component of fungal cell walls and the arthropod exoskeleton [1]. The renewable polymer is composed of β-1,4-linked N-acetyl-d-glucosamine (GlcNAc) monomers. Chitinases (EC 3.2.1.14) hydrolyze the bonds between GlcNAc residues. Due to their involvement in defence reactions against pathogens, earlier studies on chitinases were mainly focused on gene clone and transformation, especially on the gene *ech-42* [2–6]. Later, Seidl *et al*. [7] reported the first genome-wide analysis of fungal chitinase genes using the *Trichoderma reesei* QM6a genome as reference, from which 18 chitinases were identified and phylogenetically divided into 3 main groups (A, B and C) that each can be further divided into several subgroups. Subsequently, the chitinase classification model of *T. reesei* was expanded to some other filamentous fungi, and the size of chitinase gene family among fungal taxa is significantly variable, ranging from 1 in *Schizosaccharomyces pombe* to 36 in *Trichoderma virens* [1, 8]. All fungal chitinases exclusively belong to glycoside hydrolase (GH) family 18 [7].

Chitinases are involved in different aspects of the life cycle of fungi, including (1) cell wall remodelling during the stages of spore germination and constriction, hyphal growth, branching and autolysis; (2) degradation of exogenous chitin presented in the hyphal cell wall or the exoskeleton of arthropods (such as insects and shrimps) to provide nutrient sources; and (3) competition with and defence against other fungi or arthropods in an aggressive pattern by killing the fungal prey first and then feeding on the dead cell contents [ attacking other fungi (mycoparasites), insects (entomopathogenic fungi) and nematodes (nematode-trapping fungi)] [1, 9, 10].

Genera of the order Hypocreales (Sordariomycetes, Ascomycota) such as *Trichoderma*, *Metarhizium* and *Beauveria* are potent mycoparasites against plant-pathogenic fungi and insect pests that cause severe damage to crops each year [11, 12]. These bio-resources have thus been exploited as biocontrol agents in agriculture, given the imperative for an eco-friendly, safe, long-lasting and effective alternative to the dependence on fungicides in modern agriculture [13, 14]. It has been demonstrated that lysis of the host cell wall is a preliminary step in mycoparasitic attack, and chitinases make critical contributions to this process [15–17].

As noted above, the variation of chitinase gene size and the diversity of their functions, as well as the prospects for their application in agriculture, raise interesting questions regarding the evolution of this important gene family. Recent studies showed that the number of group B and group C chitinases were increased in mycoparasitic fungal species and their amino acid sequences showed low levels of conservation, which indicated that chitinases have gone through diversifying evolutionary trajectories [9, 18]. However, some key issues remain to be addressed, i.e. although group B and group C chitinases were predicted to be mycoparasitic-related, the specific roles played by the two groups remain poorly understood; and evidence for the adaptive molecular evolution of fungal chitinases also needs to be established.

This study is designed to address the above-mentioned issues by using genomic information for 72 representative species in Ascomycota. Specifically, the aim of the current study is to present a comprehensive insight into the phylogenetic relationships among chitinase genes, to investigate their expansion and contraction, to explore whether these evolutionary changes were driven by natural selection, and to interpret the interaction between chitinases and the mycoparasitic lifestyle.

## Methods

### Organisms used in this study

In order to provide a comprehensive understanding of chitinase evolution trajectories in Ascomycota, in the species investigated we tried to cover a relatively broad range of taxonomic groups, from rudimentary to advanced, and to include the most current information available. Genomic data for 72 representative species belonging to 41 orders of 10 classes in Ascomycota were selected based on the following criteria: (1) the taxonomic status and concept of the investigated species are clear and have been accurately identified; (2) the assembled scaffold number of a high-quality genome is less than 200 in principle and most of the genomes are annotated using the JGI annotation pipeline. The genomic data and the translated gene products were downloaded from JGI Mycocosm (https://genome.jgi.doe.gov/mycocosm/home) or the National Center for Biotechnology Information (NCBI) (https://www.ncbi.nlm.nih.gov/); detailed information is given in Table S1.

### Chitinase gene identification

The translated gene products were screened for the presence of chitinases using Position-Specific Iterated blast (PSI-blast) [19, 20] with a maximum of seven iterations and a reporting e-value threshold of 10^−3^, and all other parameters using default values; the chitinases of three *Trichoderma* model strains, *T. atroviride* IMI 206040, *T. reesei* QM6a and *T. virens* Gv29-8, were used as queries (Text S1); the blast results were retrieved, and redundancy sequences were removed. As the chitinase domain has sequence similarity with many other proteins, conserved domains were identified using the Conserved Domain Database (CDD) [21, 22] by searching against the Pfam v30.0 database with an expected threshold value of 0.001; only proteins with the GH family 18 (GH18) domain were confirmed as fungal chitinases and used for further analysis.

### Phylogenetic analysis

Owing to insufficient similarity between some chitinase members, phylogenetic analysis was carried out based on the GH18 catalytic domain of amino acid sequences [7]. The catalytic domains were aligned using MAFFT version 7.0 [23] on the MAFFT server (https://mafft.cbrc.jp/alignment/server/) with the FFT-NS-i iterative refinement method. A maximum-likelihood (ML) phylogenetic tree of concatenated alignment was generated using online PhyML 3.0 (http://www.atgc-montpellier.fr/phyml/) with Smart Model Selection [24]; an akaike information criterion (AIC) model was selected, and nearest-neighbour interchange (NNI) was chosen as the type of tree improvement.

### Chitinase gene family expansion and contraction

A maximum-parsimony (MP) phylogenetic tree of the 72 representative Ascomycota species with *Postia stiptica* and *Rhizopus microsporus* as out-group taxa was constructed based on sequences of LSU, SSU and RPB2 retrieved form GenBank or newly generated in this work (Text S2) via the program PAUP 4.0b10 [25]. Based on the MP dendrogram generated, a calibrated species tree was constructed using the software r8s v1.7.1 [26], assuming that the Devonian ascomycete *Palaeopyrenomycites devonicus* [27] represents an estimated age of 600 million years old [28, 29]. Finally, we used the likelihood approach implemented in Computational Analysis of gene Family Evolution (CAFE) to analyse the evolution of the size of gene family that evolved at rates of gain or loss [30], where a family-wide *P*-value <0.01 and a branch/node Viterbi *P*-value <0.05 was considered to be a signature of expansion or contraction for a specific gene family and specific species, respectively.

### Selection pressure analysis

A large number of homologous chitinase genes were generated because of the variety of gene sizes and the diversity of their functions. Proteinortho [31] was used to infer orthology groups of chitinases. Orthology groups including more than five species were picked for positive selection pressure analysis. The catalytic domain of each chitinase orthology group was aligned using the MAFFT server as described above, and then it was used to guide nucleic acid coding sequence alignments via PAL2NAL (http://www.bork.embl.de/pal2nal/) [32].

CodeML implemented in PAML v4.9 [33] was used to estimate nonsynonymous (*d*_N_) and synonymous (*d*_S_) substitution rates *ω* (*d*_N_/*d*_S_) and to detect positive selection in chitinase coding genes. For our purposes, four types of site models that allow the *ω* to vary among sites were used to fit to each orthology group. There were models that allow positive selection (M3: discrete, M8: beta and *ω*>1) and model that do not (M0: null, M7: beta). Each model is given a log-likelihood score lnL and the double difference between pairs of models [2*(lnL1−lnL0)=2ΔL] can be tested by performing two likelihood ratio tests (LRTs), which were compared with a χ^2^ distribution to test whether he individual *ω* was statistically different [34]. We performed two LRTs (M0 vs M3 and M7 vs M8) to determine whether there is variation in *ω* across chitinase codons and to test for evidence of positive selection as recommended by the user manual or references [35]. The site model in CodeML assumed a single *ω* among all lineages for a given site of a given topology, which lacks power in detecting positive selection that occurs at a few time points and affects a few amino acids [36]. Although the branch site model of CodeML provided a possible approach to such episodic selection, *a priori* specification of the foreground branches is required [33], which is tedious and arduous n terms of computational tractability, especially for large datasets [37].

For the reasons outlined above, more flexible and sensitive frameworks, relative to CodeML, implemented in the Datamonkey web server (http://www.datamonkey.org/), were further employed for pervasive and episodic positive selection detection [38]. To ensure confidence in identification results, we employed multiple approaches to conduct positive selection at each codon separately using fixed-effects likelihood (FEL), internal branch fixed-effects likelihood (IFEL) [39], the mixed-effects model of evolution (MEME) [40] and fast unconstrained Bayesian approximation (FUBAR) [41]. To avoid an excessive false-positive rate, sites under selection were only considered to be acceptable when they could be detected by at least two methods and were statistically significant (*P*<0.1 for MEME/FEL/IFEL and posterior probability; >0.9 for FUBAR). The adaptive branch-site random effects likelihood method (aBS-REL) [42] was used to identify individual branches with a proportion of sites under episodic positive selection.

## Results

### Biomining ascomycota genome for chitinases

Chitinases, present in the genome of each representative species, were identified using a PSI-blast strategy with fungal chitinases as the query, and the GH18 module of chitinases was further confirmed as described in the Methods section. Overall, 950 putative chitinases were identified from the 74 genomes (Table 1, Text S3). In terms of chitinase number, there were considerable variations among Ascomycota species, ranging from 1 in *Schizosaccharomyces pombe* to 42 in *Microdochium trichocladiopsis* and *Chaetosphaeriaceae* sp. The number of chitinases is <20 in most species; 31 species are in the range of 10–20, 29 species have fewer than 10, and only 14 species possess more than 30.

**Table 1.** Number of chitinases in different fungal species

No.	Species	Chitinases	No.	Species	Chitinases
1	*Trichoderma reesei*	19	38	*Lentithecium fluviatile*	13
2	*Trichoderma atroviride*	27	39	*Herpotrichia* sp.	19
3	*Trichoderma virens*	33	40	*Polyplosphaeria fusca*	21
4	*Sordaria brevicollis*	11	41	*Massarina eburnea*	11
5	*Fusarium tricinctum*	28	42	*Ascosphaera apis*	5
6	*Niesslia exilis*	18	43	*Phialophora mustea*	23
7	*Metarhizium anisopliae*	25	44	*Exophiala spinifera*	5
8	*Beauveria bassiana*	20	45	*Cyphellophora europaea*	5
9	*Escovopsis weberi*	16	46	*Aspergillus fumigatus*	18
10	*Cornipulvina* sp.	11	47	*Lobaria pulmonaria*	10
11	*Phaeoacremonium minimum*	11	48	*Umbilicaria pustulata*	6
12	Chaetosphaeriaceae sp.	42	49	*Golovinomyces cichoracearum*	6
13	*Coniochaeta ligniaria*	15	50	*Meliniomyces variabilis*	21
14	*Cryptodiaporthe* sp.	16	51	*Lachnellula subtilissima*	13
15	*Cytospora chrysosperma*	13	52	*Marssonina brunnea*	8
16	*Glomerella cingulata*	26	53	*Loramyces macrosporus*	23
17	*Lindra thallasiae*	7	54	*Botrytis cinerea*	9
18	*Magnaporthe oryzae*	17	55	*Bulgaria inquinans*	10
19	*Melanospora tiffanyae*	6	56	*Thelebolus microsporus*	10
20	*Microascus trigonosporus*	13	57	*Arthrobotrys oligospora*	16
21	*Myceliophthora similis*	13	58 58	*Ascodesmis nigricans*	2
22	*Phaeoacremonium* sp.	15	59	*Morchella snyderi*	4
23	*Khuskia oryzae*	21	60	*Tricharina praecox*	4
24	*Biscogniauxia nummularia*	13	61	*Sarcoscypha coccinea*	6
25	*Pestalotiopsis sp*.	15	62	*Peziza echinospora*	3
26	*Microdochium trichocladiopsis*	42	63	*Xylona heveae*	8
27	*Phyllosticta citrichinaensis*	6	64	*Saccharomyces cerevisiae*	2
28	*Melanops tulasnei*	4	65	*Vanderwaltozyma polyspora*	2
29	*Dissoconium aciculare*	18	66	*Arthroascus fermentans*	22
30	*Delphinella strobiligena*	6	67	*Candida albicans*	4
31	*Gloniopsis* sp.	10	68	*Clavispora lusitaniae*	2
32	*Jahnula aquatica*	12	69	*Trichomonascus petasosporus NRRL*	7
33	*Microthyrium microscopicum*	8	70	*Schizosaccharomyces japonicus*	1
34	*Myriangium duriaei*	14	71	*Schizosaccharomyces pombe*	1
35	*Mytilinidion resinicola*	9	72	*Protomyces lactucaedebilis*	4
36	*Patellaria atrata*	5	73	*Postia stiptica*	16
37	*Alternaria alternata*	14	74	*Rhizopus microsporus*	11

### Phylogenetic relationships of the ascomycota chitinases

**Fig. 1.**
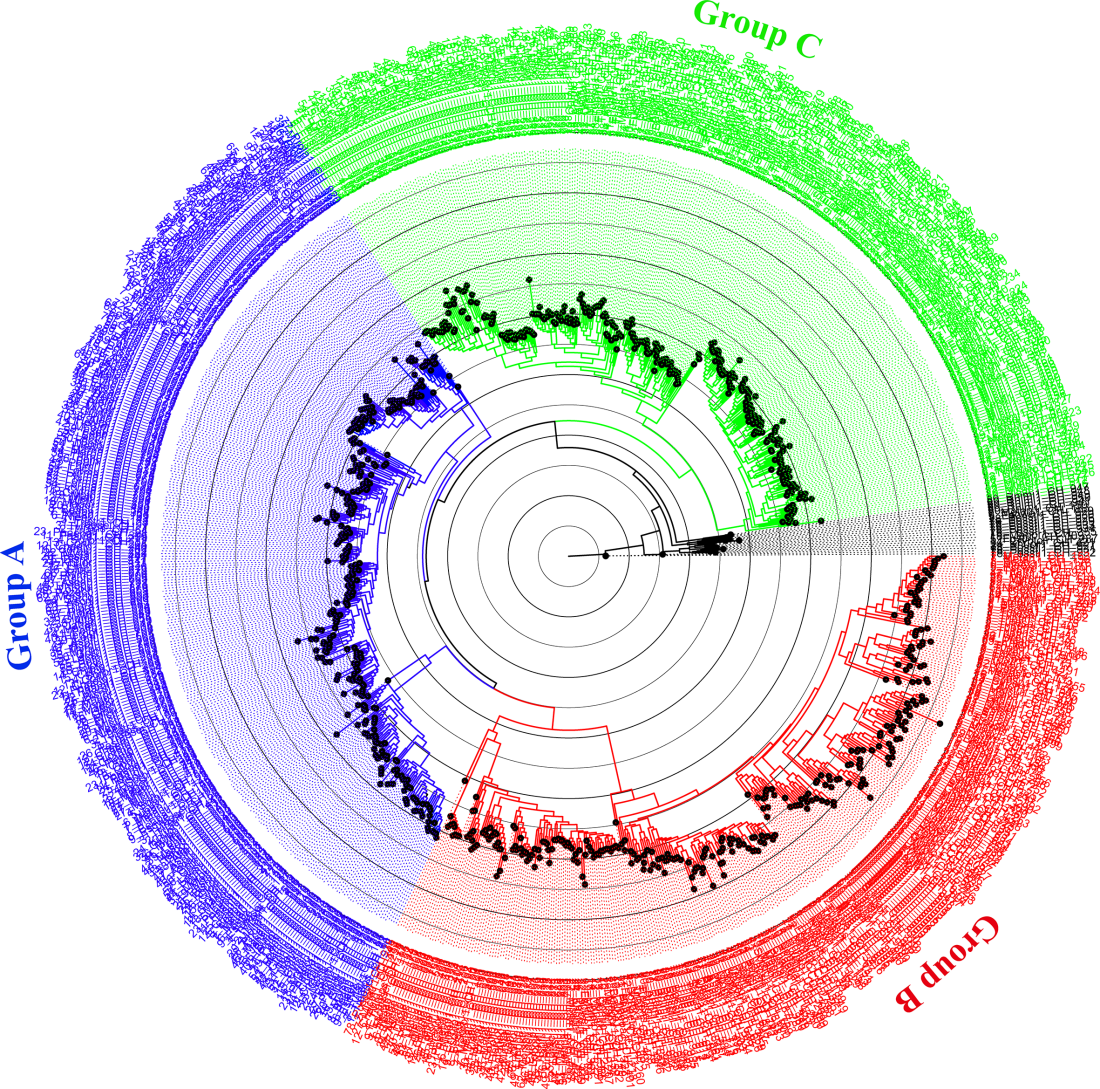
Maximum-likelihood tree showing the phylogenetic relationships of the chitinase family among 72 ascomycete species. For clarity, an expanded Newick tree is provided in Text S8.

Because of insufficient similarity due to the large variations in length and domain structure of fungal chitinases, the GH18 catalytic domain was selected for phylogenetic analysis [1, 7]. Based on the GH18 models (Text S4), 950 chitinases were divided into 3 major groups, A, B and C (Fig. 1, Text S8), and each can be further divided into subgroups.

**Fig. 2.**
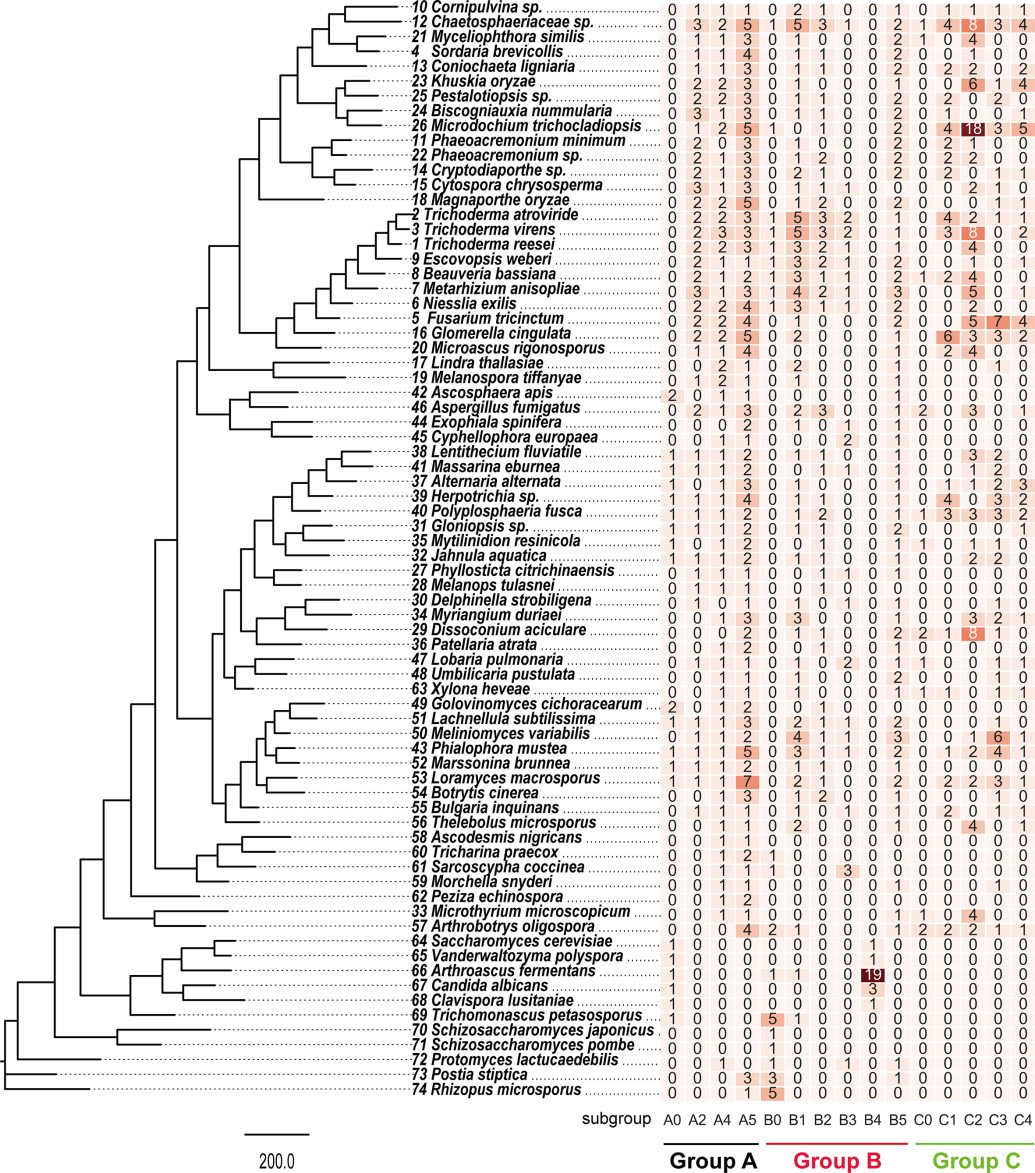
Number of fungal chitinases in different groups. The value in each square indicates the number of chitinases; the deeper the square’s colour, the higher the chitinase number.

Group A chitinases were divided into three separate subgroups, A2, A4 and A5 (Fig. S1, available in the online version of this article), which are equivalent to A-II, A-IV and A-V described previously [1, 7, 9]. Most of the species in Pezizomycotina contain 1 to 2 subgroup A2 chitinases, except Pezizomycetes, which has none (Fig. 2); species of Sordariomycetes usually have two subgroup A4 chitinases, while the remaining taxa of Pezizomycotina generally contain a single chitinase of this subgroup. The number of subgroup A5 chitinases did not show an obvious difference among species in divided. The lower fungi, Taphrinomycotina and Saccharomycotina, at the basal positions of the phylogenetic tree (Fig. 2), hardly contain any of the above-mentioned three subgroups, exept for one subgroup A4 chitinase in *Protomyces lactucaedebilis* and a few scattered lineages (indicated as A0).

Group B chitinases formed five subgroups, namely B1 to B5 (Fig. S2). Species of Hypocreales, except *Fusarium tricinctum*, possessed an average of 9.6 group B chitinases (Fig. 2), while taxa in several lineages also contained a large number of group B chitinases, e.g. Chaetosphaeriaceae sp. (12), *Meliniomyces variabilis* (9) and *Phialophora mustea* (7), but the others had many fewer (3 to 4). It is worth noting that subgroup B4 chitinases were specific for Saccharomycotina.

Group C chitinases were subdivided into subgroups C1 to C4 (Fig. S3), in which C3 and C4 are newly recognized. The number of group C chitinases displayed obvious variations within and among classes (Fig. 2). Species that code more than 10 group C chitinases included Chaetosphaeriaceae sp. (20), *Microdochium trichocladiopsis* (30), *T. virens* (13), *Fusarium tricinctum* (16), *Glomerella cingulata* (14), *Polyplosphaeria fusca* (12), *Dissoconium aciculare* (12) and *Khuskia oryzae* (11).

### Expansions and contractions of chitinase gene family in Ascomycota

**Fig. 3.**
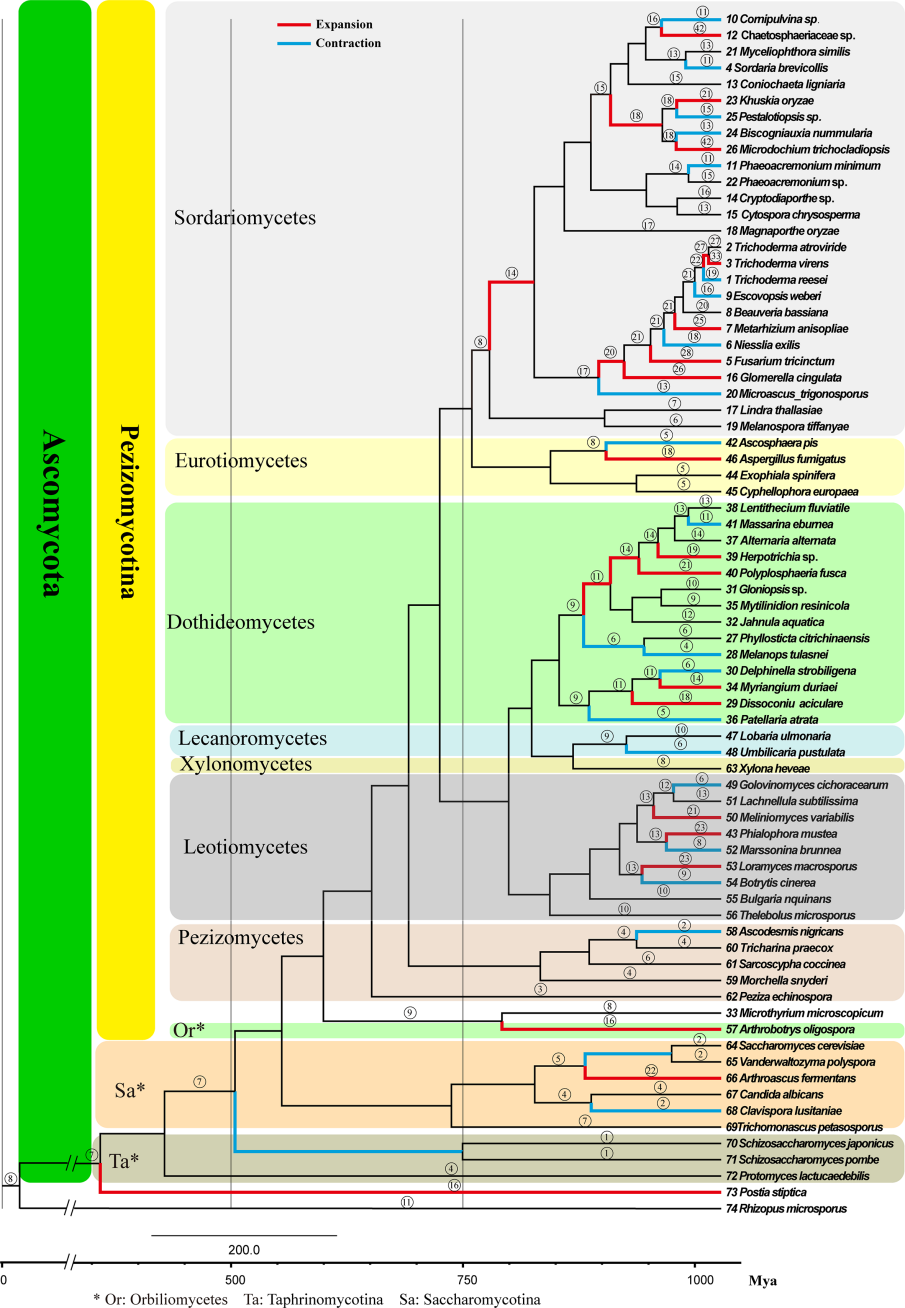
Gene expansion and contraction of chitinases in different species of Ascomycota. Numbers with a circle show the total number of GH18 genes in extant species or estimated for ancestral species and branches in red and blue indicate where significant expansion and contraction of GH18 gene family occurred, respectively.

The evolution of chitinase gene family was investigated using a random birth and death process to model gene gain and loss for each branch of the phylogenetic tree. The analysis showed that the fungal chitinase gene family evolved non-randomly (family-wide *P*-value=0.005), with branches and nodes showing significant expansions and contractions (Fig. 3).

Very few chitinase genes existed in the basal groups such as Taphrinomycotina and Saccharomycotina, where most taxa contained up to seven, except *Trichomonascus petasosporus* (Fig. 3). Contraction occurred at the node (internal branch) leading to Taphrinomycotina. Different evolutionary trajectories were exhibited among species in Saccharomycotina where contraction happened in the terminal branch leading to *Clavispora lusitaniae* and the node leading to *Vanderwaltozyma polyspora* and *Saccharomyces cerevisiae*; and expansion occurred in the correlated lineage of *Arthroascus fermentans*.

For the higher fungi, the chitinase gene family expanded or contracted only once in the species of Orbiliomycetes, Leotiomycetes, Lecanoromycetes and Pezizomycetes. However, multiple occurrences of expansion or contraction were detected in the majority species of Sordariomycetes and Dothideomycetes. The analysis showed that the gene family of all the selected species of Dothideomycetes has gone through expansions or contractions in its common ancestor or extended lineages. For example, the gene family has expanded three times in *Herpotrichia* sp. and *Polyplosphaeria fusca* during their evolution, expanded twice in the evolution history of *Lentithecium fluviatile* and *Alternaria alternata*, contracted twice in *Melanops tulasnei*, and expanded twice then contracted once in *Massarina ebumea*.

The chitinase gene family of the species in Sordariomycetes experienced more diversified evolutionary trajectories. The branch leading to Hypocreales and Diaporthales expanded 200–250 million years ago (Mya), which was ~100 Mya earlier than the expansions or contractions that occurred in other classes of Pezizomycotina. This gene family has expanded four times in the evolutionary process of *T. virens*, expanded three times in that of six species (*K. oryzae*, *M. trichocladiopsis*, *T. virens*, *F. tricinctum*, *M. anisopliae* and *G. cingulata*), expanded twice and then contracted once in that of five species (*N. exilis*, *E. weberi*, *T. reesei*, *B. nummularia* and *Pestalotiopsis* sp.), and expanded or contracted no more than twice in the other groups.

### Positive selection analysis

Owing to variation of gene size and divergence of gene function, a large number of homologous chitinase genes were generated, including orthologues and paralogues, while only the orthologues were suitable and applied to evolutionary analysis [43]. Thus, the orthologous chitinase genes were first identified prior to selection pressure analysis. Eighty-two orthology groups were recognized among the 950 chitinase genes (Table S2), and 31 of them shared by more than 5 species were picked for selection pressure analysis (Text S5 and S6). The CodeML program in the PAML package [33] was used to test whether any of these sites were subjected to positive selection in each orthology group [35] by performing two pairs of site models (M0 vs M3 and M7 vs M8). The results of the CodeML analysis suggest that amino acid sites in 12 of the 31 groups were under positive selection (Text S7), while the sites of the rest 19 groups were under purifying or neural selection.

In this study, the CodeML program was employed to examine the existence of amino acid sites on adaptive molecular evolution (*d*_N_/*d*_S_>1) rather than for specific positive site detection, since the CodeML site model is a very stringent test and lacks power to identify sites that are under episodic selection [33, 44]. Instead, more flexible and sensitive frameworks [38] were employed for pervasive and episodic positive selected codon detection, as discussed by Tomasco *et al.* [37] and Mira *et al.* [45] (see the Methods section for details). The detection results are shown in Table 2, where MEME detected the most positive selection amino acid sites, followed by IFEL and FEL, while FUBAR detected the fewest.

**Table 2.** List of codons within chitinases under positive selection pressure

Chitinase	MEME	IFEL	FEL	FUBAR
CH-376	40 (0.07) 57 (0.06) **154** (0.03) **156** (**0.09**) 301 (0.08) **330** (0) 342 (0.08)	144 (0.028) **154** (0.087) **156** (0.06) 311 (0.047) **330** (0.022)	**156** (0.067)	
Chi18-7	**126** (0.06)	106 (0.09) **126** (0.018)		
Chi18-5	88 (0.05) **264 (0) 279** (0.08) **321** (0.05) 349 (0.02)	121 (0.06) 166 (0.094) **264 (0.013) 321** (0.007)	**264** (0.007) **279** (0.075) **321** (0.089)	**264** (0.995) **279** (0.973)
Chi18-6	199 (0.03) 323 (0.08)	**245** (0.053) 297 (0.018) 362 (0.098)	**245** (0.092)	**245** (0.925)
CH-105	**193** (0.02) 218 (0.01) 213 (0.02) **291** (0)	**193** (0.051) **291** (0)	**193** (0.086) **291** (0.024)	**291** (0.936)
Chi18-rel2	12 (0.06) **102** (0.05) 103 (0.03) 279 (0.01) **321** (0.06)	264 (0.099) **321** (0.021)	**102** (**0.036**)	**102** (0.935) 218 (0.947)
Chi18-4	**186** (0.07)	25 (0.051) 38 (0.048) **186 (0.078) 400** (0.097)	**400** (0.097)	
Chi18-15	**19 (0.01) 151** (0.01) 190 (0.08)	**19** (0.008) 103 (0.085) **151** (0.036)		
Chi18-8	9 (0) 220 (0) 281 (0) 355 (0.07)			
Chi18-12	**29** (0.06) 142 (0.09) 233 (0.09) 312 (0) **366** (**0.04**)	**29** (0.009) 126 (0.099) 190 (0.057) 279 (0.088) **366** (0.046)		
CH-152	62 (0.06) 105 (0.05) 108 (0.01) 254 (0.07) 287 (0.06) 292 (0.02)	32 (0.062) 65 (0.04) 258 (0.025) 271 (0.078) 272 (0.057)		
Chi18-2	**11** (0.02) 144 (0) **255** (0.08)	3 (0.058) **11** (0.008) 146 (0.016) 191 (0.013) 212 (0.013)	**255** (0.098)	
Chi18-10	9 (0.04) 67 (0.07)	86 (0.088) 102 (0.085) 219 (0.075)		
TVC4	369 (0.08)	22 (0.052) 23 (0.078) 71 (0.034) 161 (0.074) 166 (0.013) 227 (0.033) 316 (0.08)		
TAC8	90 (0) **177** (0.09)	155 (0.061) 271 (0.051)	164 (0.061) **177** (**0.069**)	
Chi18-13	14 (0.02) 163 (0.07) 174 (0.05) **176** (0.05) 200 (0.05) **211** (0.01) 326 (0.04)	138 (0.071) **176** (0.065) **211** (**0.051**)		36 (0.925)
Chi18-16	**164** (0.03) 303 (0) 313 (0.02)	48 (0.078) 69 (0.022) 111 (0.021) 150 (0.062) **164** (0.083) 256 (0.011) 268 (0.04)		
Chi18-11	**155 (0.01) 229** (0.09) 223 (0) 276 (0) **286** (0.08)	248 (0.004) 317 (0.057)	**229** (0.025)	**155** (0.945) 189 (0.911) **229** (0.979) **286** (0.934)
CH-193	6 (0.08) 12 (0.1) 23 (0.07) 74 (0.06) 95 (0.07) 204 (0.02) **212** (0.09) 263 (0.05) 272 (0.05)	**76** (0.021) 80 (0.041) 136 (0.032) 153 (0.036) 162 (0.036) 326 (0.066) 352 (0.014)	**76** (0.084) **212** (0.069)	
TAC2	**18** (0.1)	**18** (0.001) 105 (0.067) 314 (0.09)		
TVC10	303 (0.08)	56 (0.024)		
CH-117		118 (0.061)		
CH-158	94 (0.01) **100** (0.06) 186 (0.03) **188** (0.02) 191 (0.03) 315 (0.08)	92 (0.002) 98 (0.003) **100** (0.026) 105 (0.053) 164 (0.057) 174 (0.06) **188** (0.004) 192 (0.002) 210 (0) 232 (0.06) 238 (0.032) 285 (0.061)		
Chi18-17	**100** (0.03) 159 (0.02) 226 (0.03) **221** (0.07) 230 (0)	37 (0.063) **100** (0.089) 173 (0.073) 191 (0.026)	**221** (0.048)	**221** (0.916)
CH-136		50 (0.009) 116 (0.074) 199 (0.1)		
CH-109	92 (0.04) **247 (0.03) 359 (0.09) 392** (0.06) 413 (0.04) 443 (0.09)	30 (0.041) 251 (0.079) 268 (0.053) 299 (0.002) 343 (0.008) **359** (0.094) 362 (0.033) 410 (0.002)	**247** (0.024) **392** (0.046)	
TVC-6	**21** (0.05) 327 (0.01)	**21** (0.033) 65 (0.08) 178 (0.049)		
CH-520	76 (0.01) 113 (0.04) 160 (0.07) 163 (0.08) 164 (0.07) 167 (0.07) 189 (0.07)			
CH-540	83 (0.08) 220 (0.02)	77 (0.013) 100 (0.086) 112 (0.044) 218 (0.007) 224 (0.099) 250 (0.072) 260 (0.033) 277 (0.09) 310 (0.005)		

*Numbers showing amino acid sites and statistical significances. Those in bold indicate sites detected by more than one method that were also statistically significant (*P*<0.1 for MEME/FEL/IFEL and posterior probability >0.9 for FUBAR).

Positive selection sites were only considered to be acceptable when they could be detected by at least two methods and were statistically significant to avoid an excessive false-positive rate. Given this premise, positive selection sites were existing in 20 orthology groups of chitinases. Specifically, 3 sites in four chitinases (Chi18-5, Chi18-11, CH-109 and CH-376) were under positive selection, 2 sites that had a significant probability were under positive selection in 10 chitinases like Chi18-2, Chi18-17 and CH-193, and 1 site evolving under positive Darwinian selection was found in the rest of the orthology groups (Table 2). A few sites can be detected by more than two methods, i.e. site 291 of CHI-105 was approved as positive by all four methods, while sites 156 of CH-376, 193 of CH-105, 245 of Chi18-6, 102 of Chi18-rel2, and 264 and 279 of Chi18-5 were proved to be positive by three methods (Table 2).

The above results suggest that most chitinase orthology groups were under positive diversifying selection, but for a specific gene, it is still indistinct whether the entire or just particular parts of species/lineages were subjected to positive selection. Hence, the adaptive branch-site random effects likelihood (aBSREL) algorithm was used to assess branches evolving under positive selection. The aBSREL analysis showed that evidence of episodic positive selection on branches of 11 chitinase groups was recognized. Twelve branches were identified in *chi18-7*, four branches in *chi18-16*, two branches in five genes (*chi18-3*, *chi18-5*, *chi18-6*, *chi18-8* and *chi18-15*), and a single branch in four genes (*chi18-2*, *TVC4*, *TAC8* and *chi18-13*).

**Fig. 4.**
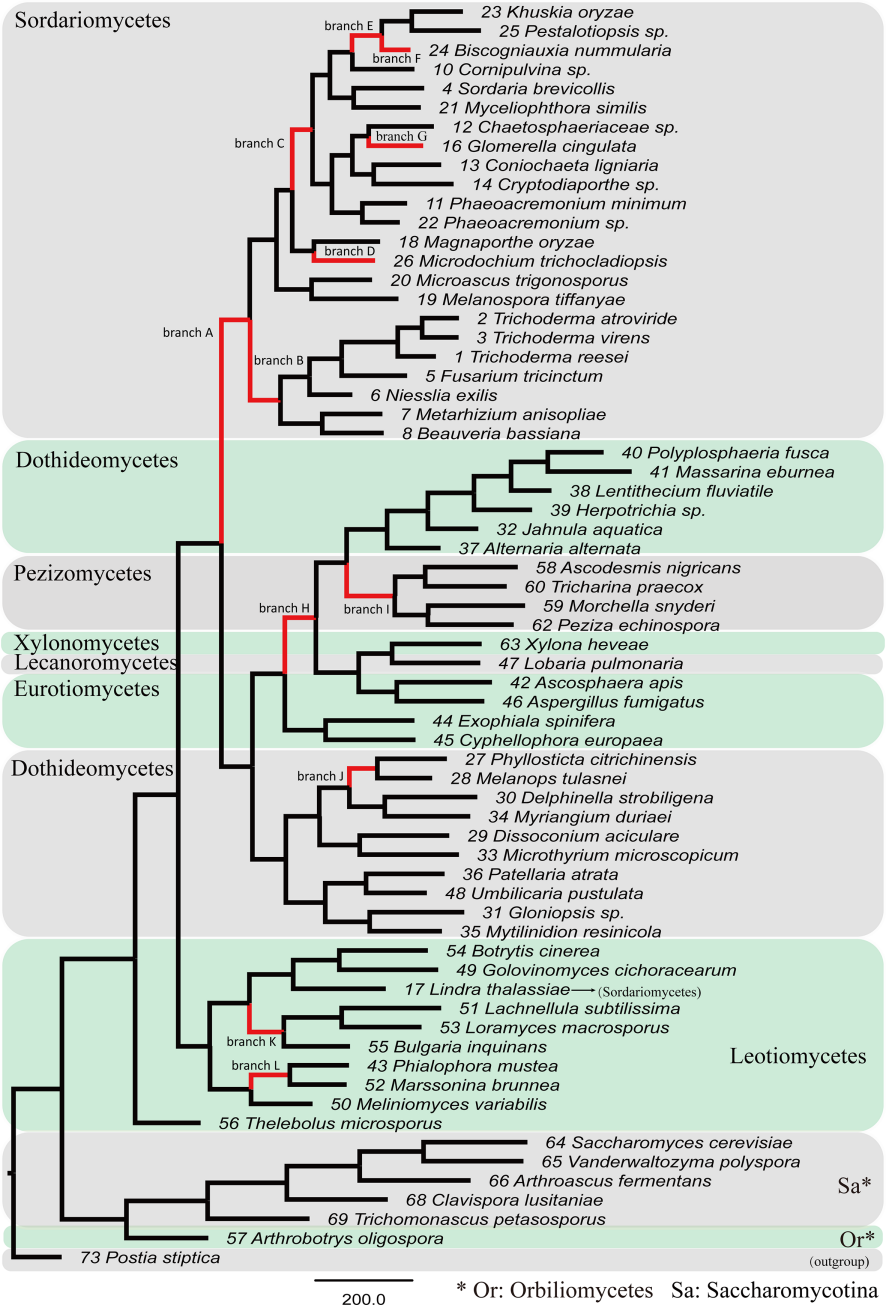
Phylogram showing chitinase gene *chi18-7* under positive selection during evolution of Ascomycota. Branches in red indicate a significant episodic positive selection.

The proportion of sites under episodic positive selection and the related selection pressure of branches/nodes are shown in Figs 4 and S4. Taking the chitinase gene group *chi18-7* as an example, this gene, quite conserved in fungal species, was identified in 66 of the 74 species. Seven of the 12 positively selected branches happened in fungi in Sordariomycetes; *chi18-7* was first subjected to positive selection in the branch leading to the class (Fig. 4, branch A), where 5.8 % of the sites were under positive selection pressure (*ω*2=21.5); then the ancestor branch of Hypocreales (Fig. 4, branch B) was undergoing adaptive molecular evolution with *ω*2=67.2 at 8.7 % of the sites. Similarly, the other five branches, including the two internal nodes (branches C and E) and the three leading to the extant species *G. cingulata* (branch G), *B. nummularia* (branch F) and *M. trichocladiopsis* (branch D), were also under positive selection (Fig. 4). In addition to Sordariomycetes, *chi18-7* was also subjected to episodic positive selection in the evolutionary history of the classes Dothideomycetes, Leotiomycetes and Pezizomycetes (Fig. 4). For the other 10 chitinase gene groups, the majority of the positively selected branches also belong to Sordariomycetes, especially Hypocreales (see Fig. S4 for details).

## Discussion

### Function discrepancy in different chitinase groups

It has been reported that group A chitinases contain a GH family 18 catalytic domain and a signal peptide in most cases [7]. Structural comparison showed that group A chitinases have a narrow substrate binding cleft and a deep substrate binding groove [9]. Seidl [10] stated that group A chitinase was ubiquitous in fungi and the estimated number varied among taxa, with an average of six for ascomycetes and four for basidiomycetes. Our results partially agree with the above conclusion, and revealed that the number of group A chitinases of ascomycetes changes along with fungal evolutionary routes; e.g. species in Saccharomycotina have ca. one, those in Pezizomycetes possess ca. two, taxa in Dothideomycetes form ca. five, and those in Leotiomycetes and Sordariomycetes recognize ca. six. Species of ascomycetes in the same class share a more or less similar number of group A chitinases. It is quite possible that the number of group A chitinases increased gradually during fungal evolution from the lower level to higher level taxa (Fig. 2).

Chi18-5 (Ech42/Cht42/Tv-Ech1) is the most studied enzyme in group A chitinases, and was first cloned from *T. harzianum* in the 1990s [5, 46]; it was then widely used as a candidate in transgenic plants to increase resistance to phytopathogens [2, 4, 6, 47]. Chi18-5 was initially deduced to be expressed during mycoparasitism, whereas recent studies indicated that it is not a mycoparasitism-specific chitinase and is also expressed during starvation and autolysis ([48]). As shown in our study, the number of group A chitinases in the mycoparasitic *Trichoderma* species, *T. atroviride* (7) and *T. virens* (8), is similar to that in the saprotrophic *T. reesei* (7), which is consistent with the work by Kubicek *et al.* [8]. As to fungi having other lifestyles or trophic types, the number of group A chitinases in two plant pathogens, *K. oryzae* (7) and *Pestalotiopsis* sp. (7), is also similar to that in *Trichoderma* species, even in the number of each subgroup. This hints that group A chitinases are probably involved in growth and development, rather than mycoparasitism.

Group B chitinases contain a GH18 module, a signal peptide at the N-terminus, and frequently a CBM1 module at C-terminus, which indicate that they are targeted to the secretory pathway and anchored to plasma membrane. Compared with group A, group B chitinases have shallower but more open substrate binding sites [9]. In most genomes of ascomycetes and basidiomycetes, two to three group B chitinases are generally present. The number of group B chitinases showed significant variation among ascomycetes. Statistical analysis showed that only 14 of the 74 representative species contain more than 5 group B chitinases (Fig. 2).

From the aspect of trophic types, it is inspiring to find that the majority of the species with more than five group B chitinases are parasitic or mycotrophic fungi. For example, among 12 of the 14 species in Pezizomycotina, *E. weberi*, *T. atroviride* and *T. virens* are mycoparasitic, *M. anisopliae* and *B. bassiana* are entomopathogenic, *P. mustea* and *M. variabilis* are plant endophytic, and *A. fumigatus* is an opportunistic human pathogen. Although humans do not have chitin, Garth *et al.* [49] showed that chitin amounts in germinating conidia and hyphae of *A. fumigatus* were significantly increased, indicating that the chitinase genes were upregulated while resting conidia transitioned to swollen conidia, and then later to hyphae. Moreover, exogenous chitinases with glycosidase activities are able to alter protein function by cleaving the glyco moieties of glycoproteins [50], and the following cascade provokes human innate immunity, which causes the host to generate a deluge of inflammatory cytokines, and this is responsible for an array of diseases, such as asthma, cystic fibrosis, cancer and autoimmune disease [50–52]. Two species in Saccharomycotina, *A. fermentans* and *T. petasosporus*, possess respectively 22 and 6 group B chitinases; *A. fermentans* shows predacious behaviour, forming penetration pegs to kill prey fungi [53, 54], while *T. petasosporus* usually parasitizes the fruiting body of *Radulomyces confluens* [55]. By contrast, the remaining 60 ascomycetes with ≤5 group B chitinases are mostly wood-decaying or saprobic. It is worth noting that plant pathogens such as *K. oryzae*, *M. oryzae*, *F. tricinctum* and *B. cinerea* have significantly lower numbers of the enzymes (an average of 3–4), which might imply that the evolution of fungal lifestyle is correlated with the expansion of group B chitinases.

With a particular interest in mycoparasites, the number of group B chitinases in the broad-spectrum mycoparasitic species, *T. atroviride* (12) and *T. virens* (12) is obviously higher than that of the obligate mycoparasite *E. weberi* (9) and saprotrophic *T. reesei* (8). Comparative genome analysis showed that the relatively low number of group B chitinases in *T. reesei* is a consequence of lifestyle transformation, switching from mycoparasitic to saprotrophic [8]. Further, our results showed that the entomopathogenic fungi *M. anisopliae* and *B. bassiana* also contain higher numbers of group B chitinases (11 and 8, respectively) than that would be expected from a random evolutionary process. This suggests that not only mycoparasitic but also entomopathogenic lifestyles have undergone selection for increased number of subgroup B chitinases, which might enable the first attack on the chitin-rich components of the cell wall of fungal prey and the exoskeleton of arthropods [9, 56].

Group C chitinases of fungi are mainly notable for their chitin-binding domain (CBM18), which is implicated in binding structurally related molecules, such as chitin. Group C chitinases show considerable similarity to theα/β subunits of the secreted zymocin killer toxin produced by the dairy yeast *Kluyveromyces lactis* [57]. In the toxin, the α-subunit contains a GH18 module that displays exochitinase activity, the α/β subunits facilitate penetration of the γ-subunit, a tRNase toxin inhibiting proliferation of *Saccharomyces*, into cytoplasm [58]. Therefore, group C chitinases are referred to as killer toxin-like chitinases, and are hypothesized to function in fungal–fungal interactions by permeabilization of fungal cell walls to allow penetration of antifungal molecules [7, 59].

Our result showed that the number of group C chitinases varies greatly among ascomycetes (Figs 2 and 3); they hardly occur in lower fungi such as Taphrinomycotina, Saccharomycotina and Pezizomycetes, while the number is greater in higher fungi, such as Sordariomycetes and Dothideomycetes. The research focused on the mycoparasitism of *Trichoderma* suggested that the saprotrophic species *T. reesei* contains only 4 group C chitinases, while the mycoparasitic *T. atroviride* and *T. virens* have significantly more (9 and 15, respectively) [8], which was confirmed by our study. Further, the entomopathogenic fungi *M. anisopliae* and *B. bassiana* also show relatively high numbers (six and seven, respectively). Based on the above, it has been deduced that a high number of group C chitinases may be correlated with the ability to adapt to/be dependent on toxic metabolites during the mycoparasitic or entomoparasitic attack. Interestingly, our results reveal that some plant-pathogens other than mycoparasitic species also have a high number of group C chitinases, such as *F. tricinctum* and *G. cingulata*, with 16 and 14, respectively. Furthermore, recent studies showed that the number of group C chitinases in mycoparasitic ascomycetes varied from 2 to 15, e.g. *E. weberi* and *Clonostachys rosea* produce 2, and *Tolypocladium ophioglossoides* has 10 [60]. Therefore, the function of group C chitinases needs further investigations, but at least they are not specifically related to mycoparasitism.

Of the 26 species in Sordariomycetes investigated, 3 are mycoparasitic and 12 are plant-pathogenic (Fig. S5). It is intriguing to note that the mycoparasitic species *T. atroviride* and *T. virens*, forming 9 and 15 group C chitinases, respectively, are able to combat a wide range of phytopathogens [61], whereas *E. weberi* with 2 group C chitinases is purely specialized on the fungal cultivar of attine ants [62]. The ancestor of *Trichoderma* was thought to be mycoparasitic, with rapid loss of group C chitinases when *T. reesei* changed lifestyle to become a saprotrophic fungus [8]. It seems that an increased number of group C chitinases is related to the host range broadening of mycoparasites. Unexpectedly, a similar pattern also appears in plant-pathogenic fungi. Three of the 12 phytopathogenic species, *F. tricinctum*, *G. cingulata* and *K. oryzae*, contain a large number of group C chitinases (16, 14 and 11, respectively). In terms of host range, *F. tricinctum* causes various plant diseases, such as *Fusarium* head blight of cereals [63] and root rot of soybean seedlings and onion bulbs [64, 65]; *G. cingulata* is the pathogen of anthracnose disease in a wide variety of temperate and sub-tropical crops [66, 67]; and *K. oryzae* causes leaf spots of *Aloe vera*, *Gossypium* sp. and *Poa pratensis* [67–69]. By contrast, the remaining nine plant-pathogenic species that have fewer group C chitinases (ranging from 2 to 4) are host-specific. For example, *Pestalotiopsis* sp. causes grey blight disease of *Persea bombycina*, *B. nummularia* damages *Carya illinoinensis* and *P. minimum* causes the loss of *Actinidia deliciosa*. Based on the above-mentioned cases, we assume that the expansion of group C chitinases is correlated with the host range broadening of mycoparasitic and plant-pathogenic fungi.

It should be pointed out that the phylogeny of genes/proteins is continually improving as more species are investigated and more sequences are added. Based on the genomics data surveyed in this study, the chitinases were clustered into groups A, B and C. Recently, Goughenour *et al*. [70] showed that group C is not resolved as distinct from group A. We are aware of that their conclusion is somewhat different from ours, and are looking forward to seeing more genomics become available for analysis.

Notably, except the chitinases that were divided into the above groups, very few with possibly bacteria-originated chitinases are not closely related to the majority of the investigated chitinases, and located at the bottom of the ML tree as scattered lineages (Fig. S2). Goughenour *et al*. [70] also demonstrated that very few bearing bacteria-originated chitinases were detected, which were shown as basal lineages in their phylogenetic tree. For example, Chi18-15, as one of the most representative proteins, was acquired through horizontal gene transfer from an actinobacterium [9]. Further homology and phylogeny analyses indicated that only 9 orthologues of Chi18-15 were identified among the 950 chitinases from 74 species that were analysed (these are located at the bottom of the ML tree) (Fig. S2).

### Chitinase gene expansions and contractions

Species of Ascomycota have a wide range of habitats and hosts, including humus, animal manure, rotten wood, plants and their residues, algae, fungi, insects and humans. The number and type of a fungal gene family result from adaption of species to the environment. Chitinase genes in most taxa have usually expanded or contracted only once in an evolutionary history, but multiple instances of expansions or contractions were detected in the majority of species of Sordariomycetes, especially Hypocreales. The evolutionary trajectory of chitinase genes reflects gene function changes of a species during evolution. *Trichoderma reesei* may serve as a case in point, whose chitinase gene family contraction was interpreted as lifestyle switching from mycoparasitic to saprotrophic [8]. *Niesslia exilis*, *F. tricinctum* and *M. anisopliae* exhibited in this study are additional examples. Their common ancestor was predicted to contain 21 chitinases; when gene expansions occurred, the plant-pathogenic *F. tricinctum* and *M. anisopliae* ended with 28 and 25 chitinases, respectively; whereas *N. exilis* switched lifestyle to saprotrophic when gene contraction occurred. Furthermore, the common ancestor of *L. subtilissima*, *G. cichoracearum* and *M. variabilis* was predicted to possess 13 chitinases; the chitinases evolved randomly in the saprotrophic *L. subtilissima* (13), while the gene family expanded in the ecdophytic *M. variabilis* (21) and contracted in *G. cichoracearum* (6) causing powdery mildews (Fig. 3). We assume therefore that expansion or contraction of chitinase gene family might be associated with lifestyle switching and broadness of host range.

### Positive selection

Gene duplication supplies raw genetic material for biological evolution [71], and subsequent divergence plays an important part in the evolution of novel gene functions [72]. Duplicated genes often form new gene families, and members of the fungal GH18 gene family were viewed as isozymes with chitinase activity [10]. As evidenced by the 31 orthology groups identified from the 950 chitinases, the evolutionary trajectories of ascomycete chitinase genes are diversified.

Positive selection plays a critical role in the functional divergence of duplicated genes, which is involved in accelerating the fixation of advantageous mutations and refining the functions of a daughter gene adopted from the ancestor [71]. Heterogeneous selection pressure detection showed that 12 chitinase groups were under positive selection, and the episodic positive selection sites and branches were detected in 20 and 11 groups, respectively (Figs 4 and S4, Table 2). This result suggests that positive selection is one of the major mechanisms driving the evolution of chitinases. It is intriguing that the majority of the positively selected branches are located in Sordariomycetes, especially in Hypocreales (Figs 4 and S4), which implies that the chitinases of this class underwent a more diversified selection. This is in accord with the results of the chitinase birth and death tests (Fig. 2), in which multiple instances of expansions or contractions occurred in the majority of Sordariomycetes species, and this also coincides with the diversity of trophic types of Hypocreales, including mycoparasitic, plant-pathogenic, saprotrophic, opportunistic and entomopathogenic (Fig. S5). Positive selection is correlated with living environment and pressure on organisms, where trophism is one of the vital factors for survival. Thus, genes related to living trophism, such as chitinase, are often under positive selection.

The number of chitinases in individual groups showed significant variations among taxa with different lifestyles and host ranges. Mycoparasites generally have a relatively high number of chitinases. Positive selection occurred on the branch leading to *Trichoderma* in chitinase gene *chi18-13* and that to *E. weberi* in *chi18-16* (Fig. S4). It has been realized that mycoparasitism is not only attributed to chitinase family members but also other hydrolytic enzymes, such as glucanases and proteases, and secondary metabolites [9]. Moreover, chitinase gene evolution is a series of events that comprise the combined actions with other functional genes. The present study provides a comprehensive analysis of chitinases in terms of phylogenetic relationship, gene expansion and contraction, and adaptive molecular evolution. Full understanding of the functions of individual chitinase genes requires all-round genomic comparisons and further biological verification.

## Conclusion

In this study, the chitinase genes from 72 representative species of Ascomycota were identified. The investigations were focused on the phylogenetic relationships among groups of chitinases, gene expansions and contractions, and molecular evolution. Our results showed that expansions and contractions of chitinase genes occurred several times during evolution, especially for the mycoparasitic species. The biological functions of the chitinase groups are different, with group B chitinases being correlated with ascomycete lifestyle evolution, while group C chitinases might be correlated with host range broadening. It has also been demonstrated that chitinases and their related amino acids sites were under episodic positive selection during Ascomycota evolution, especially at the nodes and branches leading to Hypocreales. This suggests that the chitinase family underwent diversified evolutionary trajectory and plays an essential role in species trophic style transformation, particularly evolution towards the mycoparasitic style.

## Supplementary Data

Supplementary material 1
