# Supplementary material for: Comparative molecular evolution of chitinases in ascomycota with emphasis on mycoparasitism lifestyle
Source: Microb Genom. 2021 Sep 13;7(9):000646. doi: 10.1099/mgen.0.000646 (PMC8715425; doi:10.1099/mgen.0.000646)
Supplement: Supplementary material 1 [file mgen-7-0646-s001.pdf]

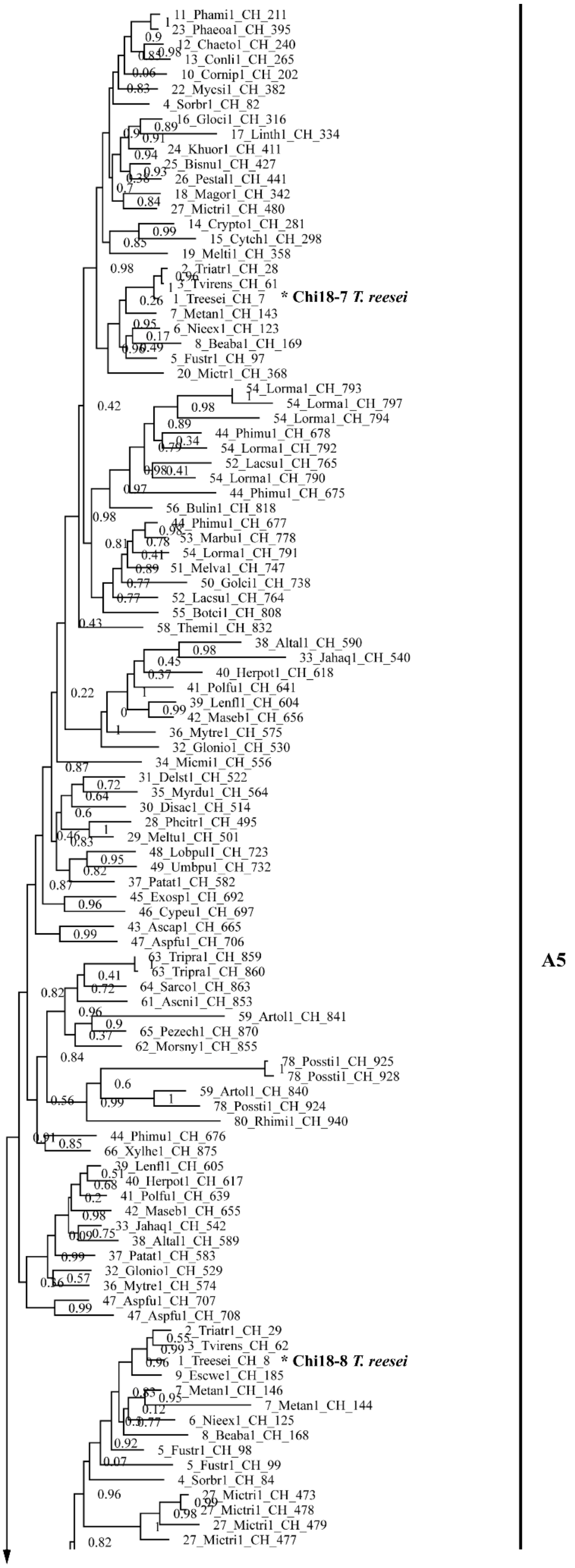
11_Phami1_CH_211
23_Phaeoa1_CH_395
12_Chacto1_CH_240
13_Conli1_CH_265
10_Cornip1_CH_202
22_Mycsi1_CH_382
4_Sorbr1_CH_82
16_Gloci1_CH_316
17_Linth1_CH_334
24_Khuor1_CH_411
25_Bisnu1_CH_427
26_Pesta1_CH_441
18_Magor1_CH_342
27_Mictri1_CH_480
14_Crypto1_CH_281
15_Cytch1_CH_298
19_Melti1_CH_358
2_Triatr1_CH_28
3_Tvirens_CH_61
1_Treesei_CH_7
* Chi18-7 T. reesei
7_Metan1_CH_143
6_Niecx1_CH_123
8_Beaba1_CH_169
5_Fustr1_CH_97
20_Mictr1_CH_368
54_Lorma1_CH_793
54_Lorma1_CH_797
54_Lorma1_CH_794
44_Phimu1_CH_678
54_Lorma1_CH_792
52_Lacsu1_CH_765
54_Lorma1_CH_790
44_Phimu1_CH_675
56_Bulin1_CH_818
44_Phimu1_CH_677
53_Marbu1_CH_778
54_Lorma1_CH_791
51_Melva1_CH_747
50_Golci1_CH_738
52_Lacsu1_CH_764
55_Botci1_CH_808
58_Themi1_CH_832
38_Alta1_CH_590
33_Jahaq1_CH_540
40_Herpot1_CH_618
41_Polfu1_CH_641
39_Lenfl1_CH_604
42_Maseb1_CH_656
36_Mytre1_CH_575
32_Glonio1_CH_530
34_Micmi1_CH_556
31_Delst1_CH_522
35_Myrdu1_CH_564
30_Disac1_CH_514
28_Phcitr1_CH_495
29_Meltu1_CH_501
48_Lobpul1_CH_723
49_Umbpu1_CH_732
37_Patat1_CH_582
45_Exosp1_CH_692
46_Cypeu1_CH_697
43_Ascap1_CH_665
47_Aspfu1_CH_706
63_Tripra1_CH_859
63_Tripra1_CH_860
64_Sarco1_CH_863
61_Ascni1_CH_853
59_Artol1_CH_841
65_Pezech1_CH_870
62_Morsny1_CH_855
78_Possti1_CH_925
78_Possti1_CH_928
59_Artol1_CH_840
78_Possti1_CH_924
80_Rhimi1_CH_940
44_Phimu1_CH_676
66_Xylhe1_CH_875
39_Lenfl1_CH_605
40_Herpot1_CH_617
41_Polfu1_CH_639
42_Maseb1_CH_655
33_Jahaq1_CH_542
38_Alta1_CH_589
37_Patat1_CH_583
32_Glonio1_CH_529
36_Mytre1_CH_574
47_Aspfu1_CH_707
47_Aspfu1_CH_708
2_Triatr1_CH_29
3_Tvirens_CH_62
1_Treesei_CH_8
* Chi18-8 T. reesei
9_Escwe1_CH_185
7_Metan1_CH_146
7_Metan1_CH_144
6_Niecx1_CH_125
8_Beaba1_CH_168
5_Fustr1_CH_98
5_Fustr1_CH_99
4_Sorbr1_CH_84
27_Mictri1_CH_473
27_Mictri1_CH_478
27_Mictri1_CH_479
27_Mictri1_CH_477
A5

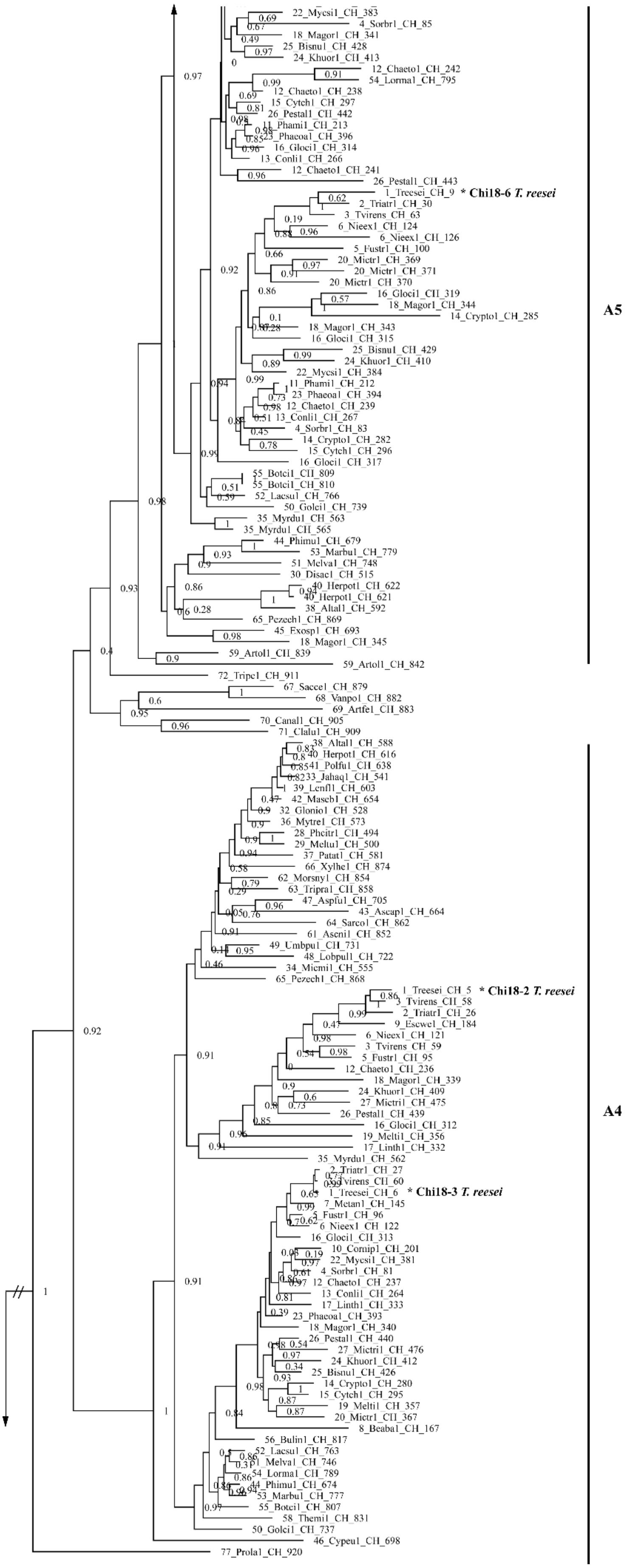
22_Mycsi1_CH_383
4_Sorbr1_CH_85
18_Magor1_CH_341
25_Bisnu1_CH_428
24_Khuor1_CH_413
12_Chaeto1_CH_242
54_Lorma1_CH_795
12_Chaeto1_CH_238
15_Cytch1_CH_297
26_Pestal1_CH_442
11_Phami1_CH_213
23_Phaeoa1_CH_396
16_Gloci1_CH_314
13_Conli1_CH_266
12_Chaeto1_CH_241
26_Pestal1_CH_443
1_Treesei_CH_9
* Chi18-6 *T. reesei*
2_Triatr1_CH_30
3_Tvirens_CH_63
6_Nieex1_CH_124
6_Nieex1_CH_126
5_Fustr1_CH_100
20_Mictr1_CH_369
20_Mictr1_CH_371
20_Mictr1_CH_370
16_Gloci1_CH_319
18_Magor1_CH_344
14_Crypto1_CH_285
18_Magor1_CH_343
16_Gloci1_CH_315
25_Bisnu1_CH_429
24_Khuor1_CH_410
22_Mycsi1_CH_384
11_Phami1_CH_212
23_Phaeoa1_CH_394
12_Chaeto1_CH_239
13_Conli1_CH_267
4_Sorbr1_CH_83
14_Crypto1_CH_282
15_Cytch1_CH_296
16_Gloci1_CH_317
55_Botci1_CH_809
55_Botci1_CH_810
52_Lacsu1_CH_766
50_Golci1_CH_739
35_Myrdu1_CH_563
35_Myrdu1_CH_565
44_Phimu1_CH_679
53_Marbu1_CH_779
51_Melva1_CH_748
30_Disac1_CH_515
40_Herpot1_CH_622
40_Herpot1_CH_621
38_Altal1_CH_592
65_Pezech1_CH_869
45_Exosp1_CH_693
18_Magor1_CH_345
59_Artol1_CH_839
59_Artol1_CH_842
72_Tripe1_CH_911
67_Sacce1_CH_879
68_Vanpo1_CH_882
69_Artfe1_CH_883
70_Canal1_CH_905
71_Clalu1_CH_909
A5
38_Altal1_CH_588
40_Herpot1_CH_616
41_Polfu1_CH_638
33_Jahaq1_CH_541
39_Lenfl1_CH_603
42_Maseb1_CH_654
32_Glonio1_CH_528
36_Mytre1_CH_573
28_Pheitr1_CH_494
29_Meltu1_CH_500
37_Patat1_CH_581
66_Xylhe1_CH_874
62_Morsny1_CH_854
63_Tripra1_CH_858
47_Aspfu1_CH_705
43_Ascap1_CH_664
64_Sarco1_CH_862
61_Ascni1_CH_852
49_Umbpu1_CH_731
48_Lobpul1_CH_722
34_Micmi1_CH_555
65_Pezech1_CH_868
1_Treesei_CH_5
* Chi18-2 *T. reesei*
3_Tvirens_CH_58
2_Triatr1_CH_26
9_Escwe1_CH_184
6_Nieex1_CH_121
3_Tvirens_CH_59
5_Fustr1_CH_95
12_Chaeto1_CH_236
18_Magor1_CH_339
24_Khuor1_CH_409
27_Mictri1_CH_475
26_Pestal1_CH_439
16_Gloci1_CH_312
19_Melti1_CH_356
17_Linth1_CH_332
35_Myrdu1_CH_562
2_Triatr1_CH_27
3_Tvirens_CH_60
1_Treesei_CH_6
* Chi18-3 *T. reesei*
7_Metan1_CH_145
5_Fustr1_CH_96
6_Nieex1_CH_122
16_Gloci1_CH_313
10_Cornip1_CH_201
22_Mycsi1_CH_381
4_Sorbr1_CH_81
12_Chaeto1_CH_237
13_Conli1_CH_264
17_Linth1_CH_333
23_Phaeoa1_CH_393
18_Magor1_CH_340
26_Pestal1_CH_440
27_Mictri1_CH_476
24_Khuor1_CH_412
25_Bisnu1_CH_426
14_Crypto1_CH_280
15_Cytch1_CH_295
19_Melti1_CH_357
20_Mictr1_CH_367
8_Beaba1_CH_167
56_Bulin1_CH_817
52_Lacsu1_CH_763
51_Melva1_CH_746
54_Lorma1_CH_789
44_Phimu1_CH_674
53_Marbu1_CH_777
55_Botci1_CH_807
58_Themi1_CH_831
50_Golci1_CH_737
46_Cypeu1_CH_698
77_Prola1_CH_920
A4

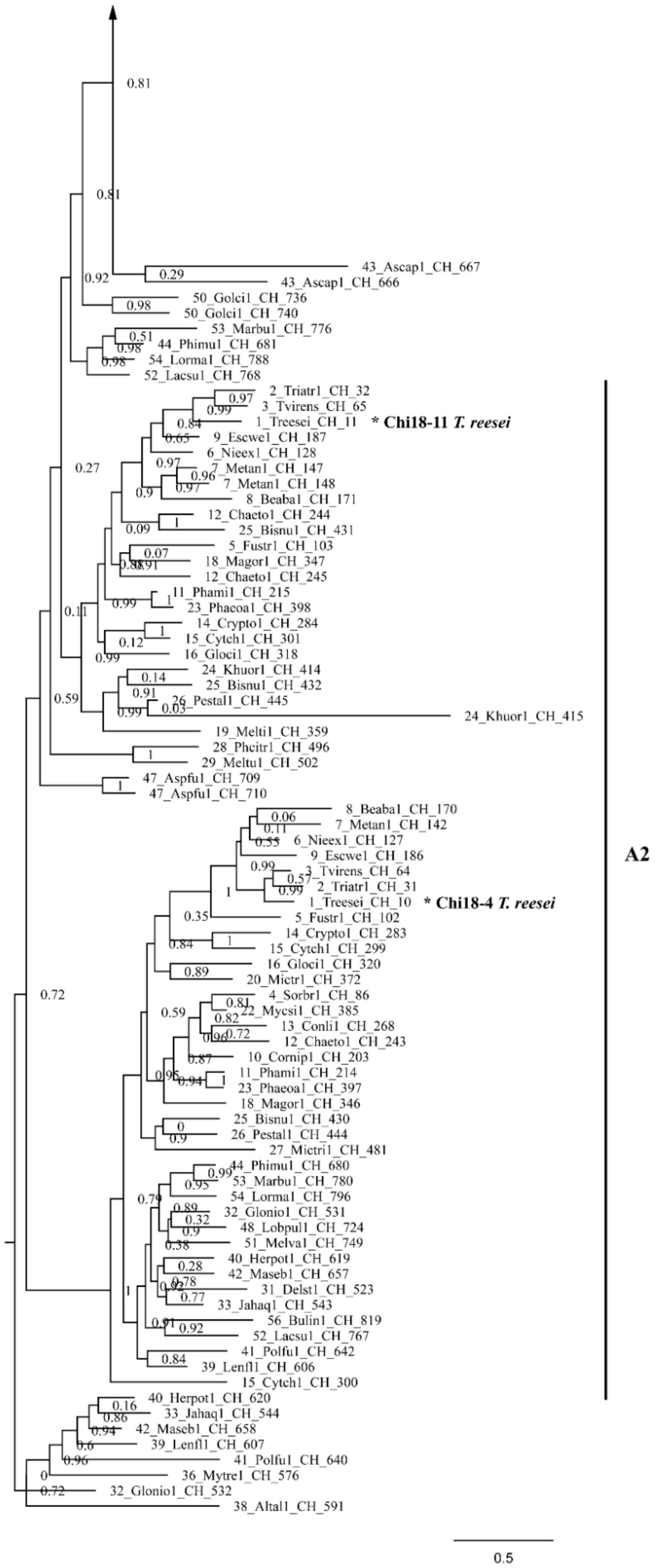

Supplementary Figure S1 Maximum-likelihood tree showing phylogeny of Group A chitinases of Ascomycota

*: Phylogenetic localization of the reported chitinases

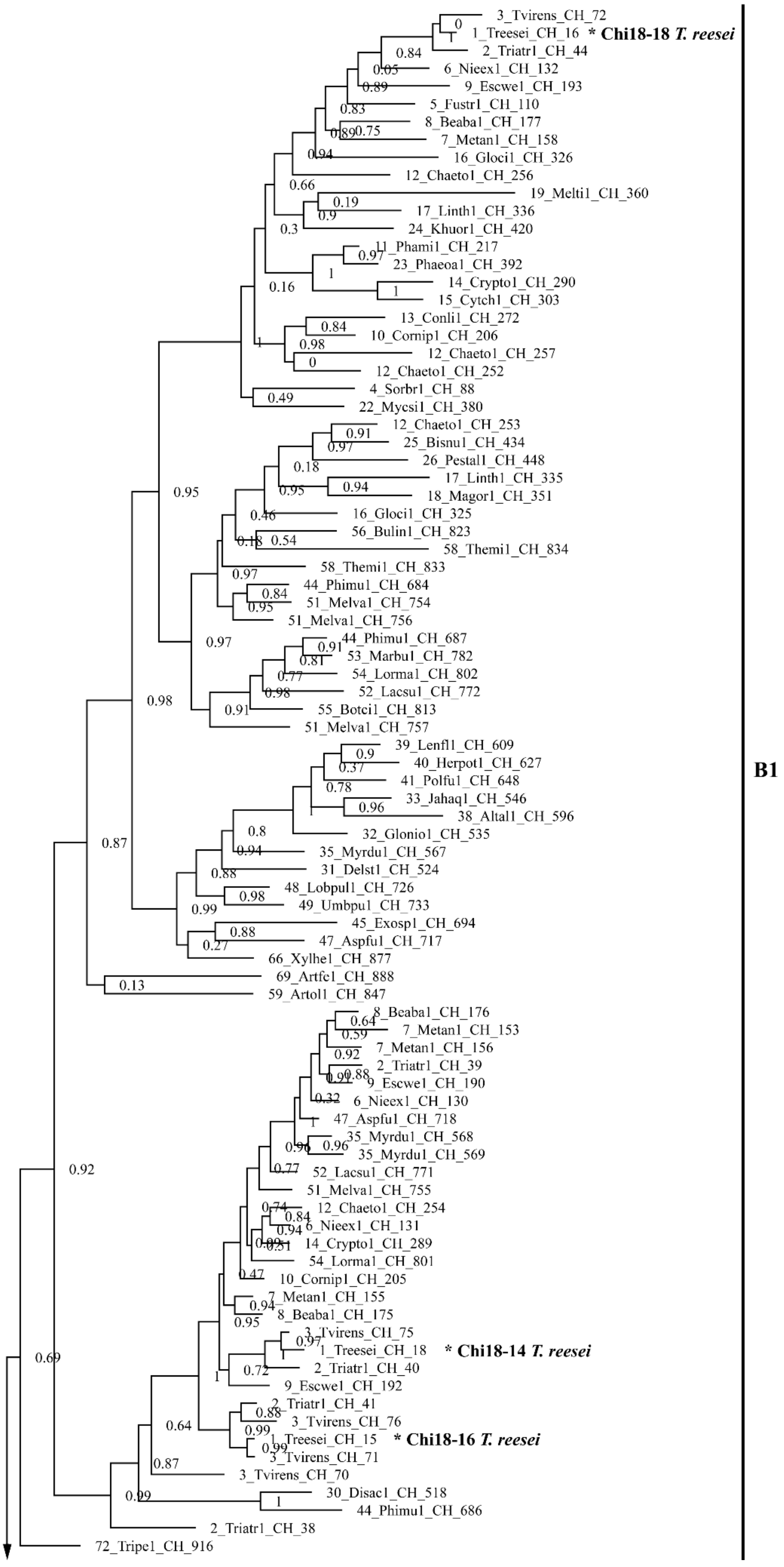
3_Tvirens_CH_72
1_Treesei_CH_16
* Chi18-18 T. reesei
2_Triatr1_CH_44
6_Nieex1_CH_132
9_Escwe1_CH_193
5_Fustr1_CH_110
8_Beaba1_CH_177
7_Metan1_CH_158
16_Gloci1_CH_326
12_Chaeto1_CH_256
19_Melti1_CH_360
17_Linth1_CH_336
24_Khuor1_CH_420
11_Phami1_CH_217
23_Phaeoa1_CH_392
14_Crypto1_CH_290
15_Cytch1_CH_303
13_Conli1_CH_272
10_Cornip1_CH_206
12_Chaeto1_CH_257
12_Chaeto1_CH_252
4_Sorbr1_CH_88
22_Mycsi1_CH_380
12_Chaeto1_CH_253
25_Bisnu1_CH_434
26_Pestal1_CH_448
17_Linth1_CH_335
18_Magor1_CH_351
16_Gloci1_CH_325
56_Bulin1_CH_823
58_Themi1_CH_834
58_Themi1_CH_833
44_Phimu1_CH_684
51_Melva1_CH_754
51_Melva1_CH_756
44_Phimu1_CH_687
53_Marbu1_CH_782
54_Lorma1_CH_802
52_Lacsu1_CH_772
55_Botci1_CH_813
51_Melva1_CH_757
39_Lenfl1_CH_609
40_Herpot1_CH_627
41_Polfu1_CH_648
33_Jahaq1_CH_546
38_Altal1_CH_596
32_Glonio1_CH_535
35_Myrdu1_CH_567
31_Delst1_CH_524
48_Lobpul1_CH_726
49_Umbpu1_CH_733
45_Exosp1_CH_694
47_Aspfu1_CH_717
66_Xylhe1_CH_877
69_Artfc1_CH_888
59_Artol1_CH_847
8_Beaba1_CH_176
7_Metan1_CH_153
7_Metan1_CH_156
2_Triatr1_CH_39
9_Escwe1_CH_190
6_Nieex1_CH_130
47_Aspfu1_CH_718
35_Myrdu1_CH_568
35_Myrdu1_CH_569
52_Lacsu1_CH_771
51_Melva1_CH_755
12_Chaeto1_CH_254
6_Nieex1_CH_131
14_Crypto1_CH_289
54_Lorma1_CH_801
10_Cornip1_CH_205
7_Metan1_CH_155
8_Beaba1_CH_175
3_Tvirens_CH_75
1_Treesei_CH_18
* Chi18-14 T. reesei
2_Triatr1_CH_40
9_Escwe1_CH_192
2_Triatr1_CH_41
3_Tvirens_CH_76
1_Treesei_CH_15
* Chi18-16 T. reesei
3_Tvirens_CH_71
3_Tvirens_CH_70
30_Disac1_CH_518
44_Phimu1_CH_686
2_Triatr1_CH_38
72_Tripe1_CH_916
B1

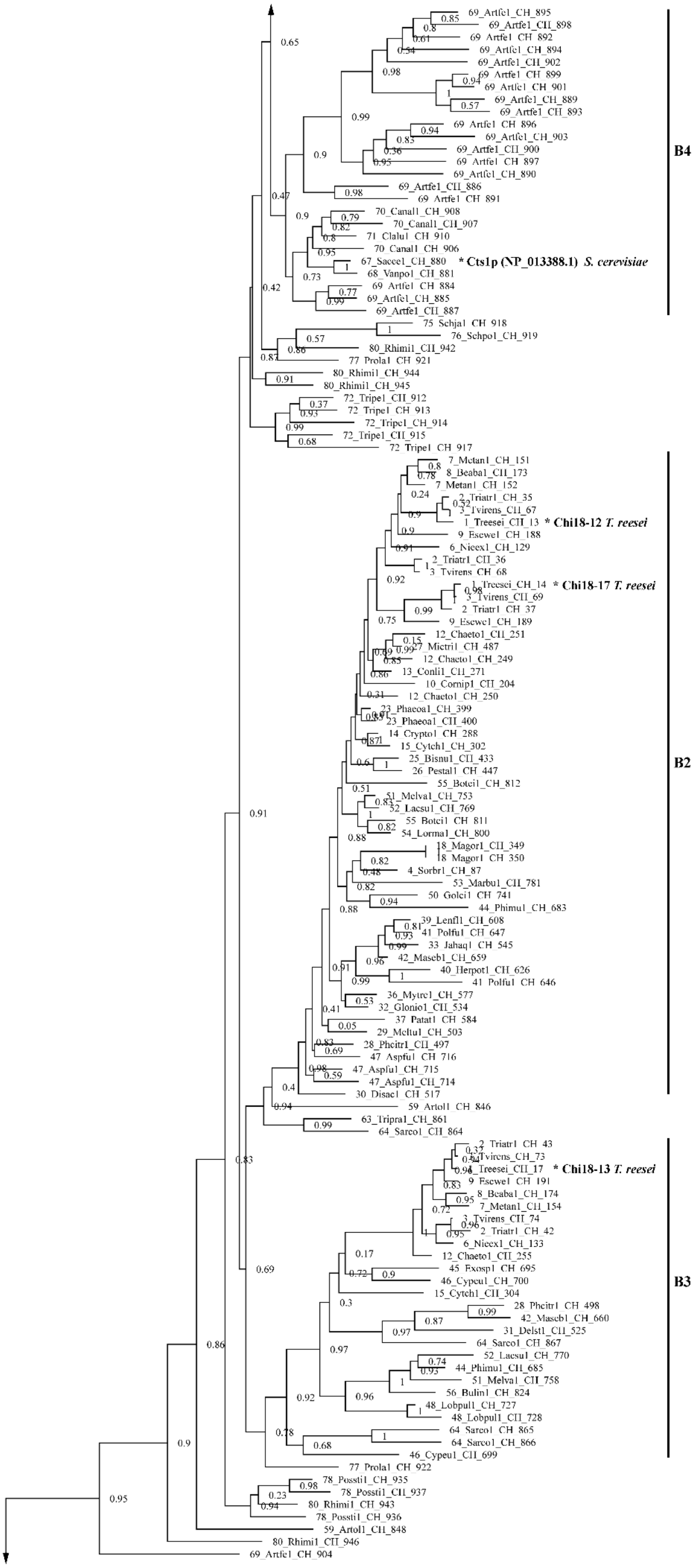

69_Artfe1_CH_895
69_Artfe1_CH_898
69_Artfe1_CH_892
69_Artfe1_CH_894
69_Artfe1_CH_902
69_Artfe1_CH_899
69_Artfe1_CH_901
69_Artfe1_CH_889
69_Artfe1_CH_893
69_Artfe1_CH_896
69_Artfe1_CH_903
69_Artfe1_CH_900
69_Artfe1_CH_897
69_Artfe1_CH_890
69_Artfe1_CH_886
69_Artfe1_CH_891
70_Canal1_CH_908
70_Canal1_CH_907
71_Clalu1_CH_910
70_Canal1_CH_906
67_Sacce1_CH_880
* Cts1p (NP_013388.1) S. cerevisiae
68_Vanpo1_CH_881
69_Artfe1_CH_884
69_Artfe1_CH_885
69_Artfe1_CH_887
B4
75_Schja1_CH_918
76_Schpo1_CH_919
80_Rhimi1_CH_942
77_Prola1_CH_921
80_Rhimi1_CH_944
80_Rhimi1_CH_945
72_Tripe1_CH_912
72_Tripe1_CH_913
72_Tripe1_CH_914
72_Tripe1_CH_915
72_Tripe1_CH_917
7_Metan1_CH_151
8_Beaba1_CH_173
7_Metan1_CH_152
2_Triatr1_CH_35
3_Tvirens_CH_67
1_Treesei_CH_13
* Chi18-12 T. reesei
9_Escwe1_CH_188
6_Nieex1_CH_129
2_Triatr1_CH_36
3_Tvirens_CH_68
1_Treesei_CH_14
* Chi18-17 T. reesei
3_Tvirens_CH_69
2_Triatr1_CH_37
9_Escwe1_CH_189
12_Chaeto1_CH_251
27_Mictri1_CH_487
12_Chaeto1_CH_249
13_Conli1_CH_271
10_Cornip1_CH_204
12_Chaeto1_CH_250
23_Phaeoa1_CH_399
23_Phaeoa1_CH_400
14_Crypto1_CH_288
15_Cytch1_CH_302
25_Bisnu1_CH_433
26_Pestal1_CH_447
55_Botci1_CH_812
51_Melva1_CH_753
52_Lacsu1_CH_769
55_Botci1_CH_811
54_Lorma1_CH_800
18_Magor1_CH_349
18_Magor1_CH_350
4_Sorbr1_CH_87
53_Marbu1_CH_781
50_Golci1_CH_741
44_Phimu1_CH_683
39_Lenfl1_CH_608
41_Polfu1_CH_647
33_Jahaq1_CH_545
42_Maseb1_CH_659
40_Herpot1_CH_626
41_Polfu1_CH_646
36_Mytre1_CH_577
32_Glonio1_CH_534
37_Patat1_CH_584
29_Meltu1_CH_503
28_Phcitr1_CH_497
47_Aspfu1_CH_716
47_Aspfu1_CH_715
47_Aspfu1_CH_714
30_Disac1_CH_517
B2
59_Artol1_CH_846
63_Tripra1_CH_861
64_Sarco1_CH_864
2_Triatr1_CH_43
3_Tvirens_CH_73
1_Treesei_CH_17
* Chi18-13 T. reesei
9_Escwe1_CH_191
8_Beaba1_CH_174
7_Metan1_CH_154
3_Tvirens_CH_74
2_Triatr1_CH_42
6_Nieex1_CH_133
12_Chaeto1_CH_255
45_Exosp1_CH_695
46_Cypeu1_CH_700
15_Cytch1_CH_304
28_Phcitr1_CH_498
42_Maseb1_CH_660
31_Delst1_CH_525
64_Sarco1_CH_867
52_Lacsu1_CH_770
44_Phimu1_CH_685
51_Melva1_CH_758
56_Bulin1_CH_824
48_Lobpul1_CH_727
48_Lobpul1_CH_728
64_Sarco1_CH_865
64_Sarco1_CH_866
46_Cypeu1_CH_699
B3
77_Prola1_CH_922
78_Posstil_CH_935
78_Posstil_CH_937
80_Rhimi1_CH_943
78_Posstil_CH_936
59_Artol1_CH_848
80_Rhimi1_CH_946
69_Artfe1_CH_904

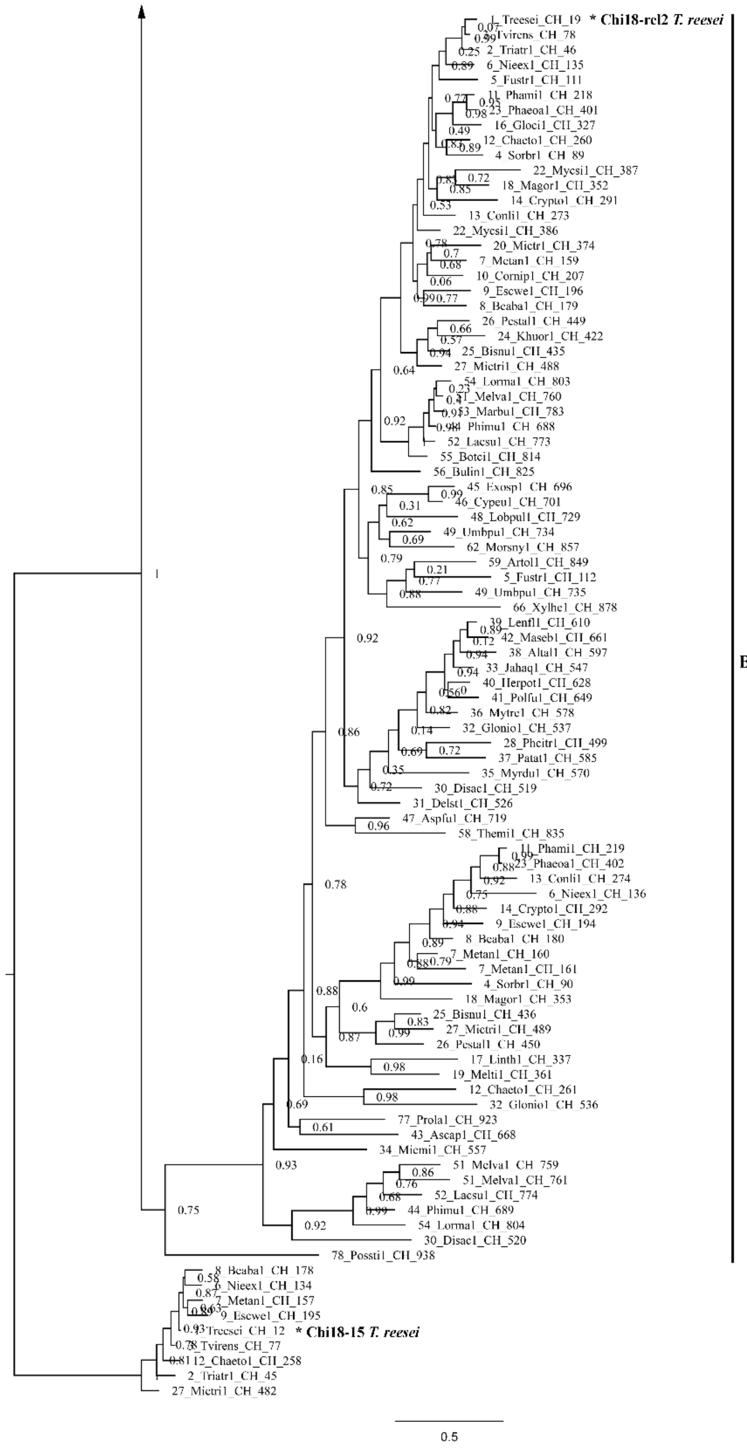

Supplementary Figure S2 Maximum-likelihood tree of Group B chitinases of Ascomycota

*: Phylogenetic localization of the reported chitinases

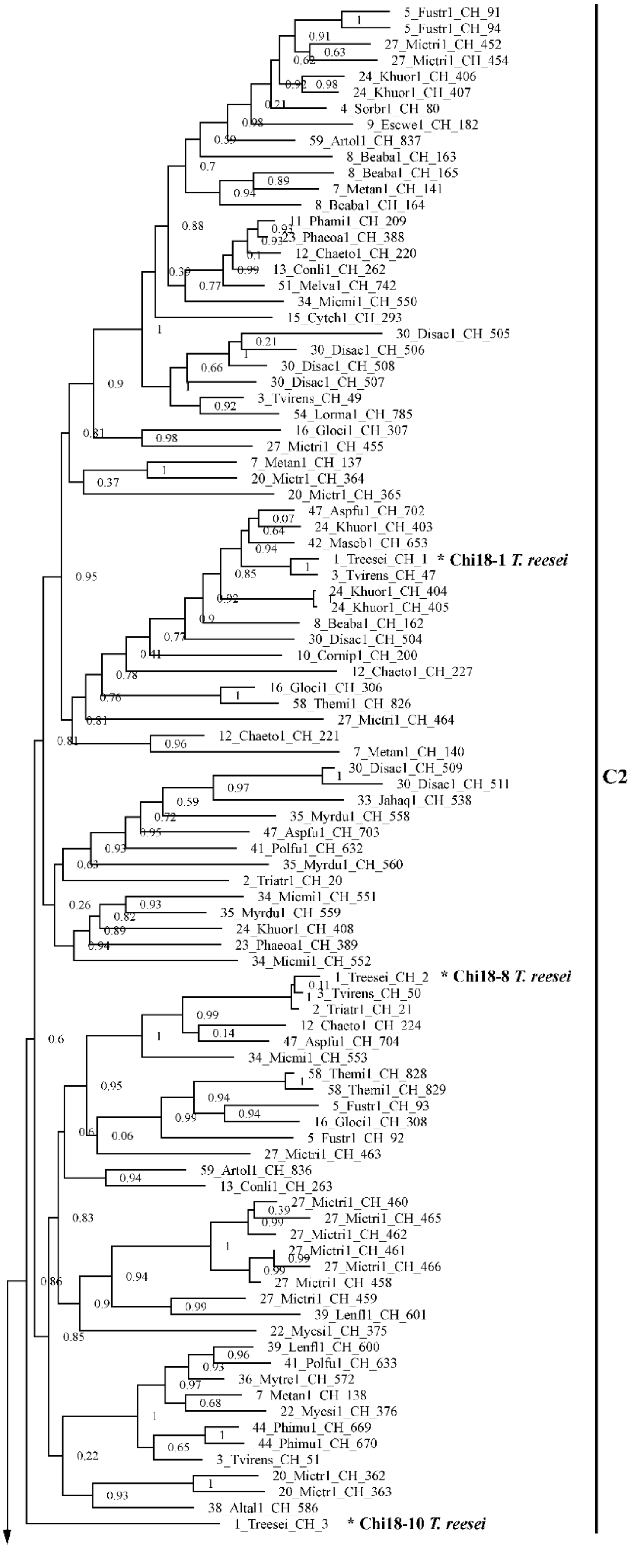
C2
* Chi18-1 T. reesei
* Chi18-8 T. reesei
* Chi18-10 T. reesei

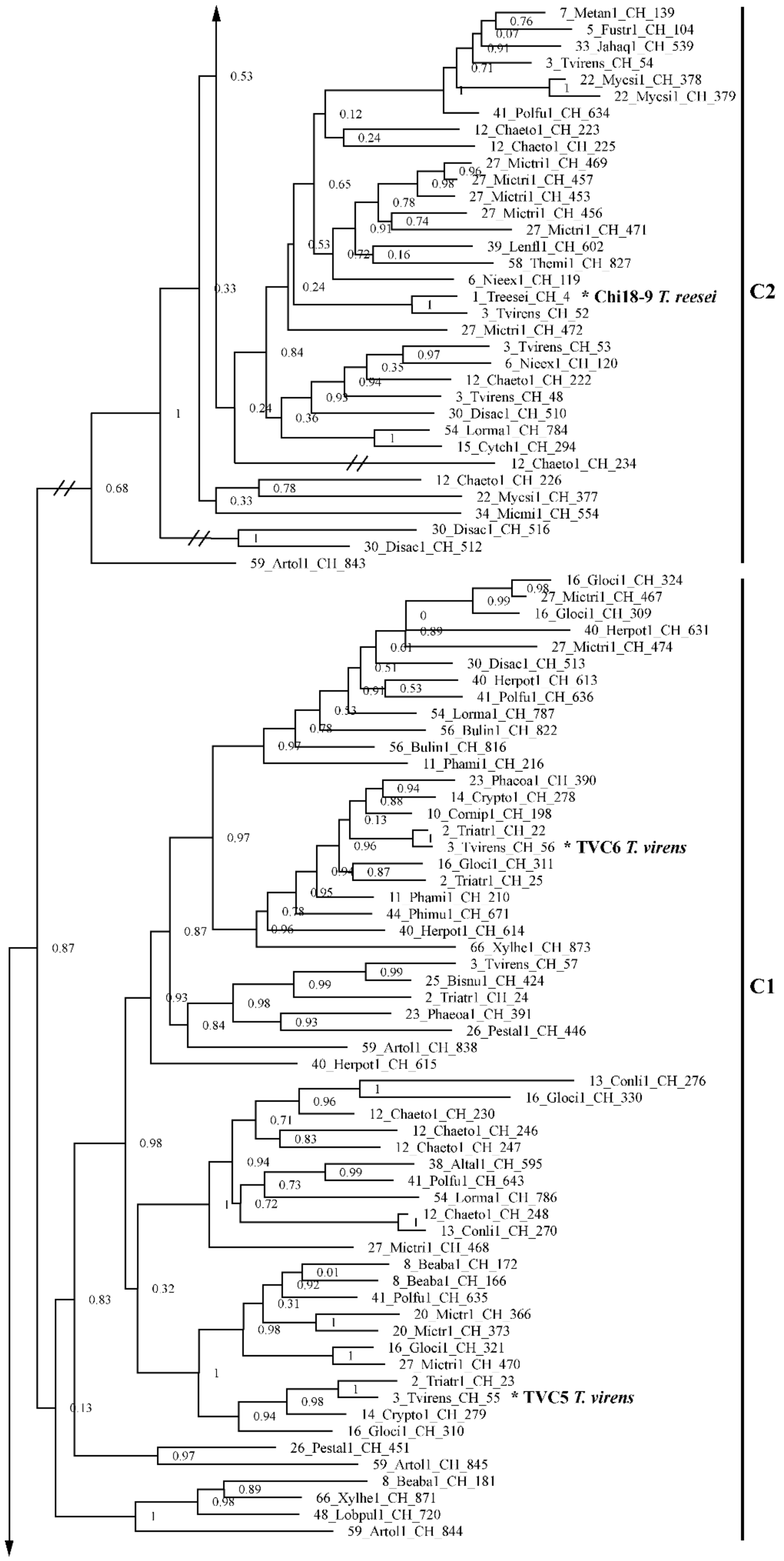
7_Metan1_CH_139
5_Fustr1_CH_104
33_Jahaq1_CH_539
3_Tvirens_CH_54
22_Mycsi1_CH_378
22_Mycsi1_CH_379
41_Polfu1_CH_634
12_Chaeto1_CH_223
12_Chaeto1_CH_225
27_Mictri1_CH_469
27_Mictri1_CH_457
27_Mictri1_CH_453
27_Mictri1_CH_456
27_Mictri1_CH_471
39_Lenfl1_CH_602
58_Themi1_CH_827
6_Nieex1_CH_119
1_Treesei_CH_4 * Chi18-9 T. reesei
3_Tvirens_CH_52
27_Mictri1_CH_472
3_Tvirens_CH_53
6_Nieex1_CH_120
12_Chaeto1_CH_222
3_Tvirens_CH_48
30_Disac1_CH_510
54_Lorma1_CH_784
15_Cytch1_CH_294
12_Chaeto1_CH_234
12_Chaeto1_CH_226
22_Mycsi1_CH_377
34_Micmi1_CH_554
30_Disac1_CH_516
30_Disac1_CH_512
59_Arto11_CH_843
C2
16_Gloci1_CH_324
27_Mictri1_CH_467
16_Gloci1_CH_309
40_Herpot1_CH_631
27_Mictri1_CH_474
30_Disac1_CH_513
40_Herpot1_CH_613
41_Polfu1_CH_636
54_Lorma1_CH_787
56_Bulin1_CH_822
56_Bulin1_CH_816
11_Phami1_CH_216
23_Phaeoa1_CH_390
14_Crypto1_CH_278
10_Cornip1_CH_198
2_Triatr1_CH_22
3_Tvirens_CH_56 * TVC6 T. virens
16_Gloci1_CH_311
2_Triatr1_CH_25
11_Phami1_CH_210
44_Phimu1_CH_671
40_Herpot1_CH_614
66_Xylhe1_CH_873
3_Tvirens_CH_57
25_Bisnu1_CH_424
2_Triatr1_CH_24
23_Phaeoa1_CH_391
26_Pestal1_CH_446
59_Arto11_CH_838
40_Herpot1_CH_615
13_Conli1_CH_276
16_Gloci1_CH_330
12_Chaeto1_CH_230
12_Chaeto1_CH_246
12_Chaeto1_CH_247
38_Altal1_CH_595
41_Polfu1_CH_643
54_Lorma1_CH_786
12_Chaeto1_CH_248
13_Conli1_CH_270
27_Mictri1_CH_468
8_Beaba1_CH_172
8_Beaba1_CH_166
41_Polfu1_CH_635
20_Mictr1_CH_366
20_Mictr1_CH_373
16_Gloci1_CH_321
27_Mictri1_CH_470
2_Triatr1_CH_23
3_Tvirens_CH_55 * TVC5 T. virens
14_Crypto1_CH_279
16_Gloci1_CH_310
26_Pestal1_CH_451
59_Arto11_CH_845
8_Beaba1_CH_181
66_Xylhe1_CH_871
48_Lobpul1_CH_720
59_Arto11_CH_844
C1

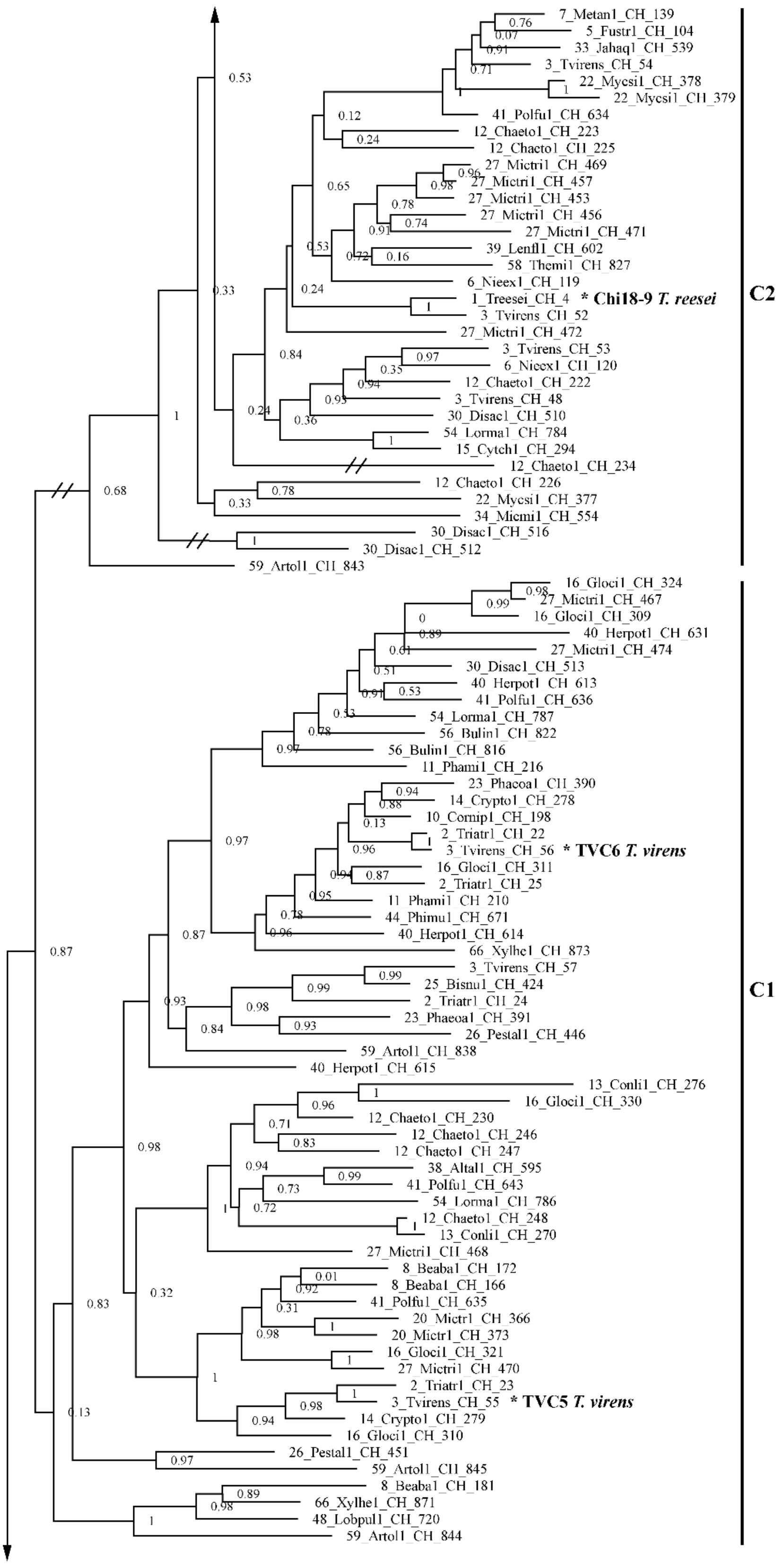

Supplementary Figure S3 Maximum-likelihood tree of Group C chitinases of Ascomycota

*: Phylogenetic localization of the reported chitinases

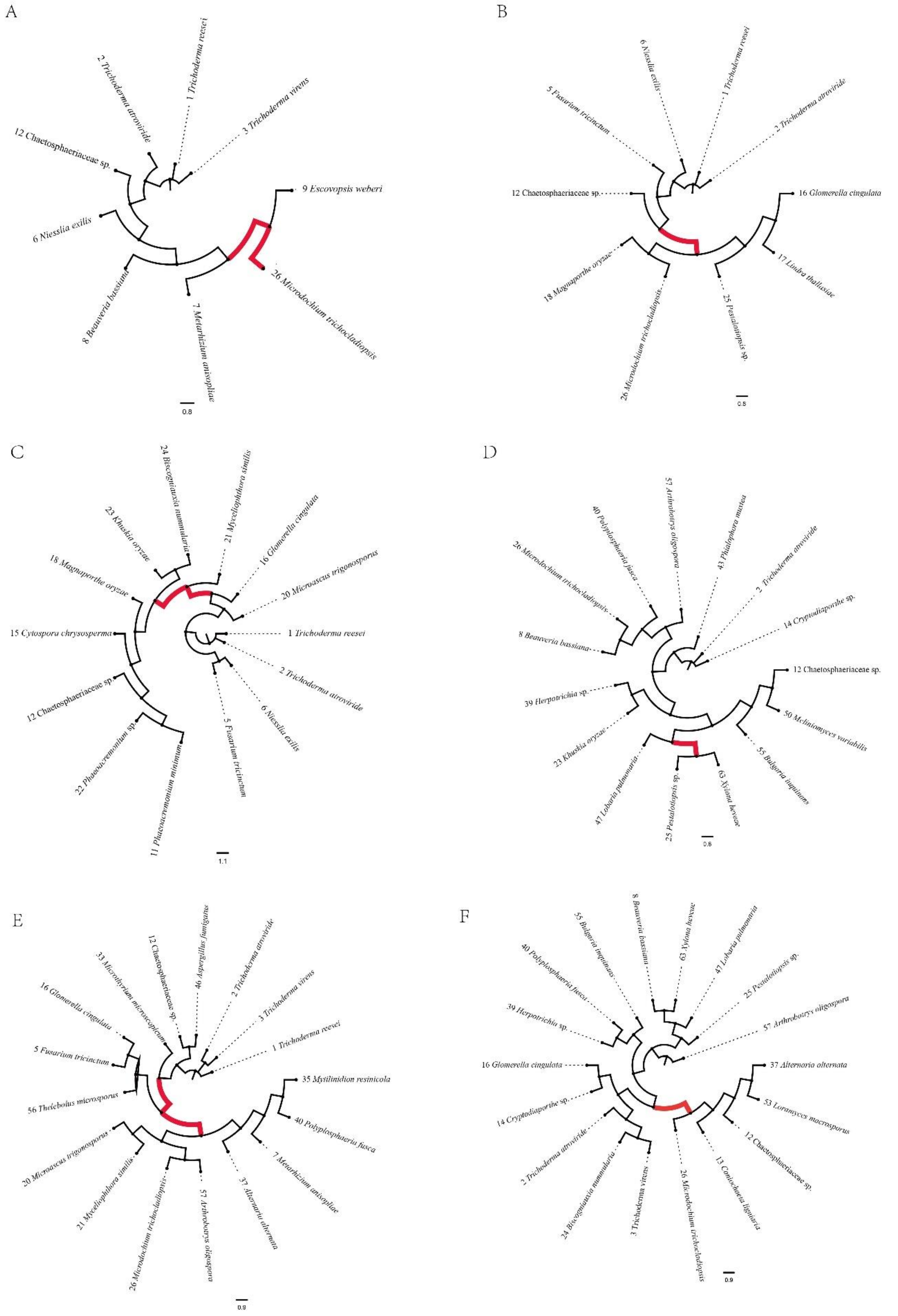
A
1 Trichoderma reesei
2 Trichoderma atroviride
3 Trichoderma virens
12 Chaetosphaeriaceae sp.
9 Escovopsis weberi
6 Niesslia exilis
8 Beauveria bassiana
7 Metarhizium anisopliae
26 Microdochium trichocladiopsis
0.8
B
6 Niesslia exilis
1 Trichoderma reesei
5 Fusarium tricinctum
2 Trichoderma atroviride
12 Chaetosphaeriaceae sp.
16 Glomerella cingulata
18 Magnaporthe oryzae
17 Lindra thalassiae
26 Microdochium trichocladiopsis
25 Pestalotiopsis sp.
0.5
C
24 Biscogniauxia nummularia
21 Myceliophthora similis
23 Khuskia oryzae
16 Glomerella cingulata
18 Magnaporthe oryzae
20 Microascus trigonosporus
15 Cytospora chrysosperma
1 Trichoderma reesei
2 Trichoderma atroviride
12 Chaetosphaeriaceae sp.
6 Niesslia exilis
5 Fusarium tricinctum
22 Phaeoacremonium sp.
11 Phaeoacremonium minimum
1.1
D
57 Arthrobotrys oligospora
40 Polyplosphaeria fusca
43 Phialophora mustea
26 Microdochium trichocladiopsis
2 Trichoderma atroviride
14 Cryptodiaporthe sp.
8 Beauveria bassiana
12 Chaetosphaeriaceae sp.
39 Herpotrichia sp.
50 Meliniomyces variabilis
23 Khuskia oryzae
55 Bulgaria inquinans
47 Lobaria pulmonaria
25 Pestalotiopsis sp.
63 Xylona heveae
0.8
E
12 Chaetosphaeriaceae sp.
46 Aspergillus fumigatus
33 Microthyrium microscopicum
2 Trichoderma atroviride
16 Glomerella cingulata
3 Trichoderma virens
1 Trichoderma reesei
5 Fusarium tricinctum
35 Mytilinidion resinicola
56 Thelebolus microsporus
40 Polyplosphaeria fusca
20 Microascus trigonosporus
7 Metarhizium anisopliae
21 Myceliophthora similis
37 Alternaria alternata
26 Microdochium trichocladiopsis
57 Arthrobotrys oligospora
0.9
F
8 Beauveria bassiana
55 Bulgaria inquinans
63 Xylona heveae
47 Lobaria pulmonaria
40 Polyplosphaeria fusca
25 Pestalotiopsis sp.
39 Herpotrichia sp.
57 Arthrobotrys oligospora
16 Glomerella cingulata
37 Alternaria alternata
14 Cryptodiaporthe sp.
53 Loramyces macrosporus
2 Trichoderma atroviride
12 Chaetosphaeriaceae sp.
24 Biscogniauxia nummularia
3 Trichoderma virens
26 Microdochium trichocladiopsis
13 Coniochaeta ligniaria
0.9

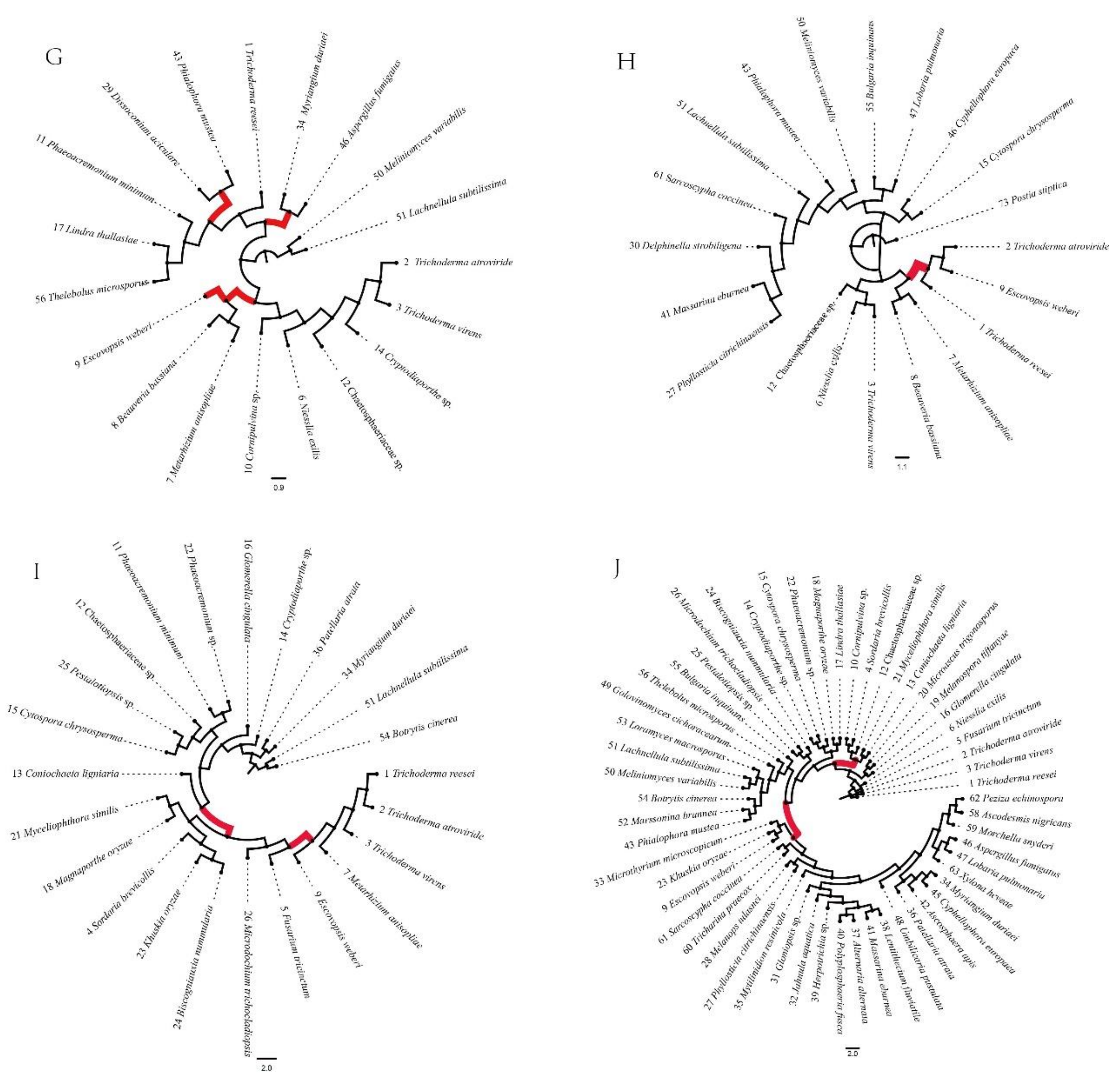

Supplementary Figure S4 Phylogram showing 10 chitinase genes under positive selection during evolution of Ascomycota. Branches in red indicating a significant episodic positive selection. A: chi18-15; B: chi18-2; C: chi18-6; D: TAC8; E: chi18-8; F: TVC4; G: chi18-16; H: chi18-13; I: chi18-5; J: chi18-3.

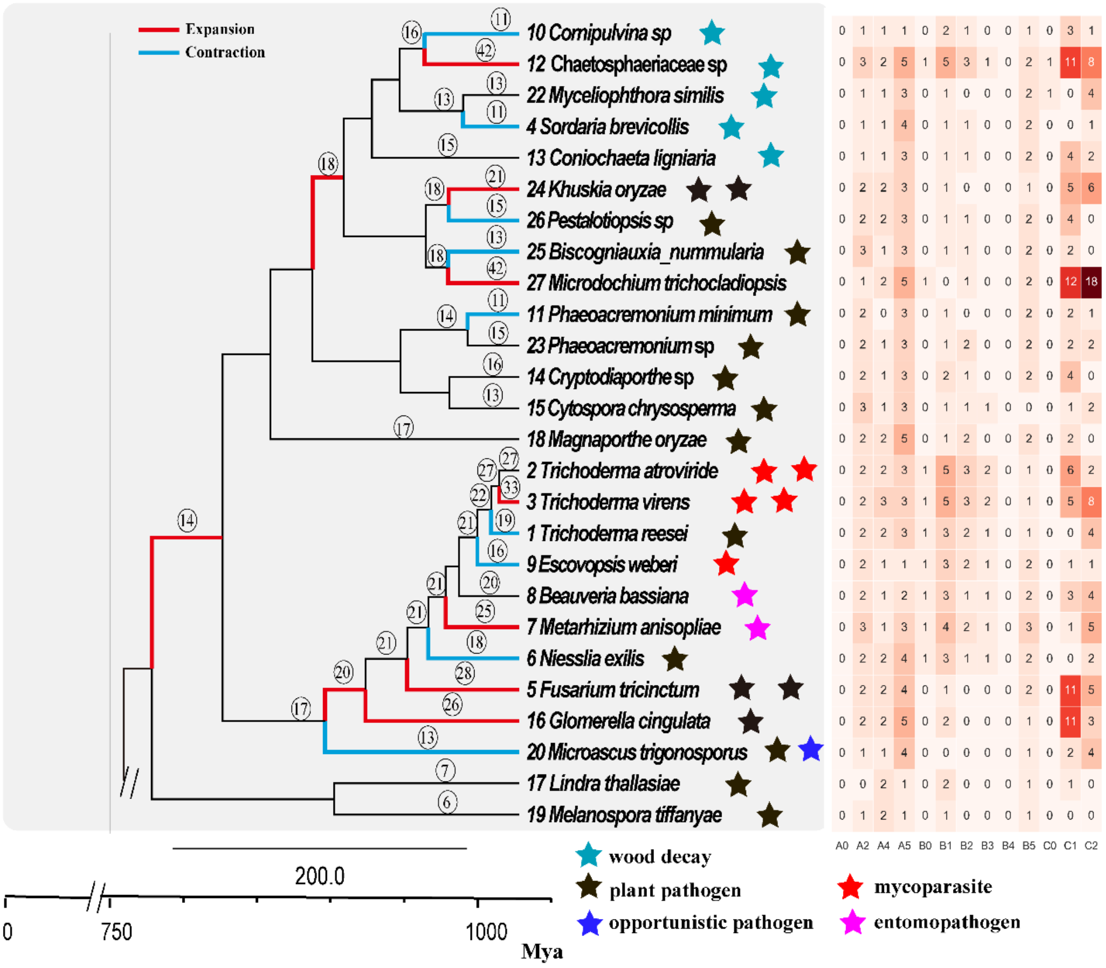

Supplementary Figure S5 Number of chitinase genes and nutrition modes of Sordariomycetes. Value in each square indicating the number of chitinases, the deeper the square's color, the higher the chitinase number.
